# Genome-Wide Association Study Reveals Marker–Trait Associations for Early Vegetative Stage Salinity Tolerance in Rice

**DOI:** 10.3390/plants10030559

**Published:** 2021-03-16

**Authors:** Ashutosh Kumar Yadav, Aruna Kumar, Nitasha Grover, Ranjith Kumar Ellur, Haritha Bollinedi, Subbaiyan Gopala Krishnan, Prolay Kumar Bhowmick, Kunnummal Kurungara Vinod, Mariappan Nagarajan, Ashok Kumar Singh

**Affiliations:** 1 Division of Genetics, ICAR—Indian Agricultural Research Institute, New Delhi 110012, India; akybio@gmail.com (A.K.Y.); grovernitasha07@yahoo.com (N.G.); ranjithellur@gmail.com (R.K.E.); haritha.agrco@gmail.com (H.B.); krish.icar@gmail.com (S.G.K.); prolaybhowmick@gmail.com (P.K.B.); kkvinodh@gmail.com (K.K.V.); 2 Amity Institute of Biotechnology, Amity University, Noida 201303, India; akumar@amity.edu; 3 Rice Breeding and Genetics Research Centre, ICAR—Indian Agricultural Research Institute, Aduthurai 612101, Tamil Nadu, India; drmnagarajan2000@yahoo.co.in

**Keywords:** rice, salt stress, salinity tolerance, SNP, GWAS, MTAs

## Abstract

Rice germplasm is a rich resource for discovering genes associated with salt tolerance. In the current study, a set of 96 accessions were evaluated for seedling stage salinity tolerance and its component traits. Significant phenotypic variation was observed among the genotypes for all the measured traits and eleven accessions with high level of salt tolerance at seedling stage were identified. The germplasm set comprised of three sub-populations and genome-wide association study (GWAS) identified a total of 23 marker–trait associations (MTAs) for traits studied. These MTAs were located on rice chromosomes 1, 2, 5, 6, 7, 9, and 12 and explained the trait phenotypic variances ranging from 13.98 to 29.88 %. Twenty-one MTAs identified in this study were located either in or near the previously reported quantitative trait loci (QTLs), while two MTAs namely, qSDW2.1 and qSNC5 were novel. A total of 18 and 13 putative annotated candidate genes were identified in a genomic region spanning ~200 kb around the MTAs *qSDW2.1* and *qSNC5,* respectively. Some of the important genes underlying the novel MTAs were *OsFBA1,* *OsFBL7*, and *mTERF* which are known to be associated with salinity tolerance in crops. These MTAs pave way for combining salinity tolerance with high yield in rice genotypes through molecular breeding.

## 1. Introduction

Rice (*Oryza sativa* L.) is cultivated in ~114 countries with an annual production of ~755 million tons [1]. It is the source of nutrition for more than three billion people across the globe. By 2050, the global population is expected to reach 9.73 billion and to feed this ever-increasing population agricultural food production has to be increased by more than 50 percent [2]. In the current scenario of shrinking land holdings, reduced availability of natural resources and changing climate, meeting the demand of food production is a daunting task. The major losses in food production are due to biotic and abiotic stresses. Salinity causes significant yield losses in rice production as it is one of the least salt-tolerant species among major cereal crops [3,4]. Generally, the concentration of salts *viz.,* sodium chloride, potassium chloride, magnesium chloride, sodium sulphate, magnesium sulphate, calcium sulphate, and sodium carbonate are high in saline soils [5]. Na^+^ and Cl^−^ are the major ions in saline soils responsible for causing injury to various biochemical and metabolic processes apart from inducing physiological drought stress in plants [4,6,7,8,9,10,11]. Rice plants can tolerate salt stress of electrical conductivity (ECe) up to 3 dSm^−1^ and for every dSm^−1^ increase, the estimated yield drop is 12% [12]. At ECe of 7.2 dSm^−1^, the yield reduction has been estimated up to 50% [13]. Therefore, to maintain global food supply, developing salt-tolerant rice varieties remains one of the primary solutions.

The seedling stage is highly sensitive for salinity stress in rice. There exists significant genetic variability for seedling stage salinity tolerance in rice and a robust high throughput screening protocol is available [14]. Salinity tolerance is a complex phenomenon both physiologically and genetically [15,16,17]. Na^+^ uptake reduction or Na^+^ exclusion, and increased K^+^ absorption to retain appropriate Na^+^/K^+^ ratio in the shoot is the typical mechanism of the salt stress tolerance in rice plants. Therefore, Na^+^, K^+^, and Na^+^/K^+^ in shoot are used as an effective criterion in determining salt stress tolerance in rice [14,18]. Furthermore, QTL mapping approach has been successful in identifying genomic regions governing salinity tolerance in rice [17,18,19,20,21,22,23,24,25,26,27]. Several QTLs have been identified, of which *Saltol* a major QTL for seedling stage salinity tolerance from landrace Pokkali was identified on chromosome 1 which regulates shoot Na^+^/K^+^ under salt stress [17,20,24]. *Saltol* is widely used in rice breeding programs aiming towards the development of varieties suited for salinity conditions [28].

To efficiently utilize the genetic diversity available for salinity tolerance in breeding programs, it is essential to detect genomic regions governing the target trait so that marker-aided breeding can be employed. Linkage analysis based QTL mapping approach pose restrictions for finding valuable natural variations in trait-associated loci due to limited variation and recombination in biparental mapping populations [29,30]. Alternatively, linkage disequilibrium (LD) based mapping is an efficient and powerful strategy to utilize germplasm for identifying MTAs [31,32]. This approach offers larger mapping resolution and ability to evaluate greater allelic diversity [33,34,35]. Association mapping was successful in identifying the genomic regions for various salt-tolerance related traits *viz.,* stress susceptibility indices of the vigor index, germination time, Na^+^ and K^+^ contents in shoot and root, net photosynthetic rate, seedling length ratio, fresh and dry weight ratio in various growth stages [36,37,38,39,40].

The present study aims at screening rice germplasm for seedling stage salinity tolerance to identify tolerant cultivars and MTAs governing salinity tolerance. These donors and MTAs can be utilized in breeding programs to develop varieties with tolerance to salinity through marker assisted selection.

## 2. Results

### 2.1. Phenotypic Evaluation

Significant phenotypic variation was observed among the genotypes for all 14 salinity tolerance related traits recorded in the current study (Supplementary Table S1). Based on salt tolerance score (STS) at EC of 13.9 dS/m, eleven genotypes namely, UPRI-2003-45, Samanta, Tompha Khau, Chandana, VLT-6, Narendra Usar Dhan II, Narendra Usar Dhan III, PMK-1, Seond Basmati, Manaswini, and Shah Pasand were tolerant with a score of 3, similar to salt-tolerant checks, FL 478, CSR 23, and CSR 27. Twenty-one genotypes were found moderately tolerant with a score 5 and remaining 61 genotypes were susceptible.

**Figure 1:**
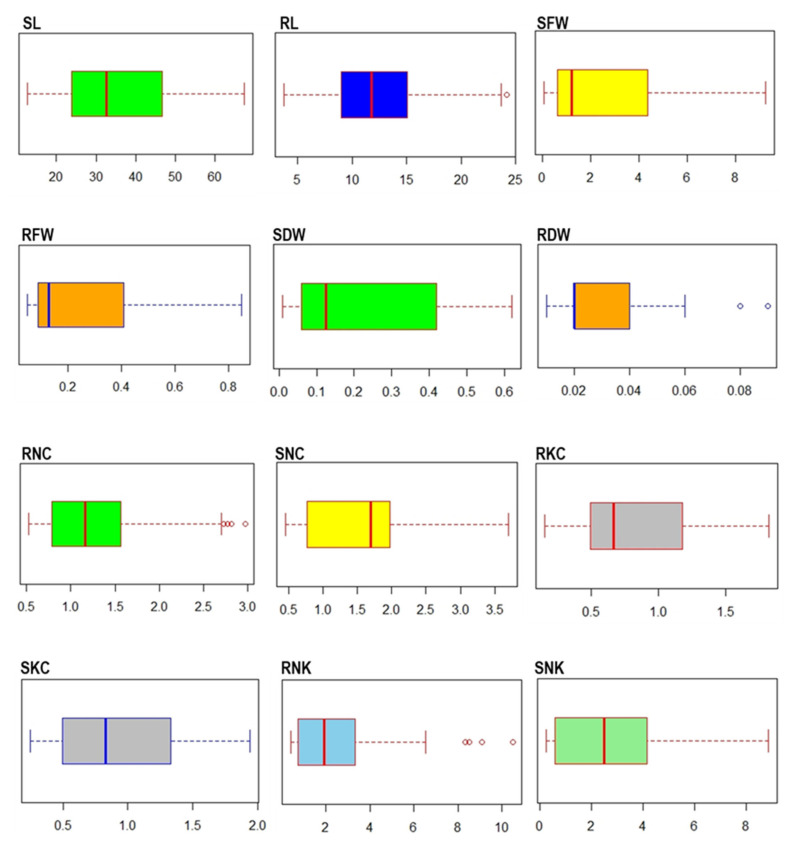
Variation in rice germplasm for traits associated with seedling stage salinity tolerance. SL, shoot length (cm); RL, root length (cm); SFW, shoot fresh weight (g); RFW, root fresh weight (g); SDW, shoot dry weight (g); RDW, root dry weight (g); RNC, root Na^+^ content (mmol/g); SNC, shoot Na^+^ content (mmol/g); RKC, root K^+^ content (mmol/g); SKC, shoot K^+^ content (mmol/g); RNK, root Na^+^/K^+^ ratio; SNK, shoot Na^+^/K^+^ ratio.

Under salt stress conditions, shoot length (SL) ranged from 12.80 cm (Pusa 1301) to 67.35 cm (Seond Basmati), with an average of 35.53 cm, while the average root length (RL) ranged from 3.75 cm (CO-51) to 24.20 cm (Tompha Khau) with an average of 12.44 cm (Figure 1). The average shoot fresh weight (SFW) was 2.51 g with minimum of 0.07 g in Tapaswani and maximum of 9.25 g in VLT-6. The average root fresh weight (RFW) was 0.24 g which ranged between 0.05 (Pusa 1490-3) to 0.85 g (Tompha Khau) (Figure 1).

Under stress conditions, shoot dry weight (SDW) ranged from 0.01 (Pusa 1301) to 0.62 g (PMK-1) with an average of 0.22 g, and root dry weight (RDW) ranged from 0.01 g (PNR381) to 0.09 g (CSR27) with an average of 0.03 g. Considerable variation between different tolerant groups was observed for Na^+^ and K^+^ content in both roots and shoots (Figure 1).

The average root Na^+^ concentration (RNC) and shoot Na^+^ concentration (SNC) was 1.30 and 1.56 mmol/g, which ranged from 0.53 mmol/g (chandana) to 2.29 mmol/g (Chimbalate Basamti) and 0.46 mmol/g (Shah Pasand) to 3.72 mmol/g (ASD 19), respectively (Figure 1). However, the highest Na^+^ concentration was seen in highly susceptible groups than others. The average root K^+^ concentration (RKC) and shoot K^+^ concentration (SKC) was 0.80 and 0.92 mmol/g, ranging from 0.16 mmol/g (Mahanadi) to 1.82 (Samanta) mmol/g and 0.24 mmol/g (Improved Shambha Mashuri) to 1.96 mmol/g (CSR23), respectively (Figure 1). Under salt stress condition the root Na^+^/K^+^ (RNK) ranged from 0.41 (Samanta) to 10.5 (Mahanadi) with an average of 2.43, while shoot Na^+^/K^+^ (SNK) ranged from 0.26 (Shah Pasand) to 8.85 (Chimbalate Basamti) with an average of 2.64 (Figure 1).

**Figure 2:**
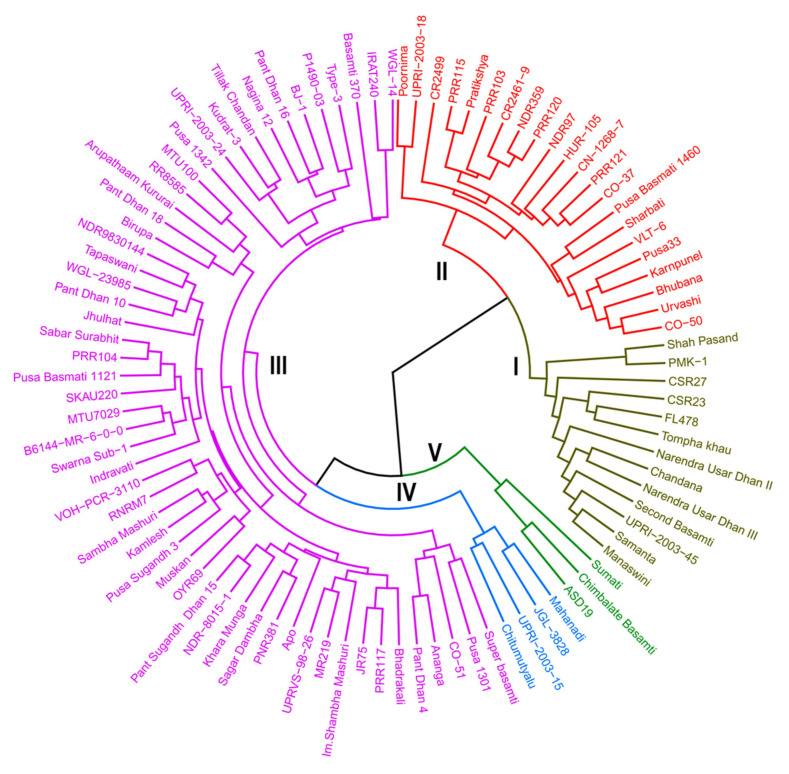
Phenogram of 96 rice genotype based on salt tolerance score and 13 morpho-physiological characters recorded under salt stress.

A dendrogram was constructed on the basis of salt tolerance score and 13 morpho-physiological characters to classify the rice genotypes. Five clusters were generated using Euclidean distance (Figure 2). Cluster I represented the tolerant genotypes along with salt tolerant check FL478, CSR 23, and CSR 27. Cluster II represented moderately tolerant genotypes except for a tolerant genotype VLT-6. Clusters III, IV, and V comprised of susceptible and highly susceptible genotypes.

### 2.2. Correlation among Traits Related to Salt Stress

**Figure 3:**
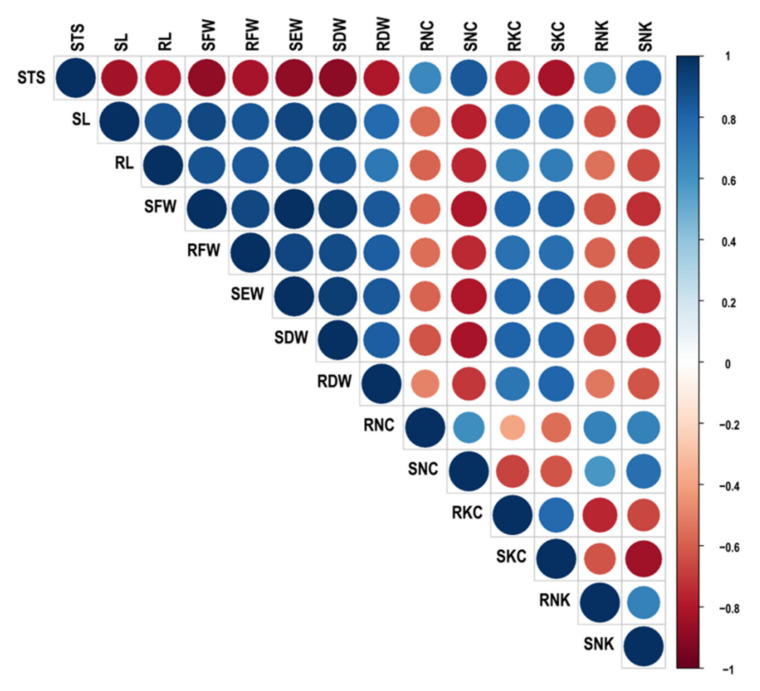
Correlation coefficients among various morpho-physiological parameters under salt stress condition. Positive correlations are displayed in blue and negative correlations in red color. Color intensity and the size of the circle are proportional to the correlation coefficients. In the right side of the correlogram, the legend color shows the correlation coefficients and the corresponding colors. STS, Salinity tolerance score; SL, shoot length (cm); RL, root length (cm); SFW, shoot fresh weight (g); RFW, root fresh weight (g); SEW, seedling weight (g); SDW, shoot dry weight (g); RDW, root dry weight (g); RNC, root Na^+^ content (mmol/g); SNC, shoot Na^+^ content (mmol/g); RKC, root K^+^ content (mmol/g); SKC, shoot K^+^ content (mmol/g); RNK, root Na^+^/K^+^ ratio; SNK, shoot Na^+^/K^+^ ratio.

For understanding the physiological traits that best define seedling stage salinity tolerance, correlation between the traits was generated (Figure 3, Supplementary Figure S1). STS Showed significant negative correlation with RKC (−0.75), SKC (−0.83), SL (−0.83), RL (−0.80), SFW (−0.89), RFW (−0.82), SEW (−089), SDW (−0.90), and RDW (−0.81). Association of STS with RNC (0.64), SNC (0.85), RNK^+^ (0.63), and SNK^+^ (0.78) were significantly positive. Na^+^ and Na^+^/K^+^ was significantly negatively correlated with length, fresh and dry weight of root and shoot, while K^+^ was significantly positively associated with all the root and shoot morphological parameters studied.

### 2.3. Population Structure

**Figure 4:**
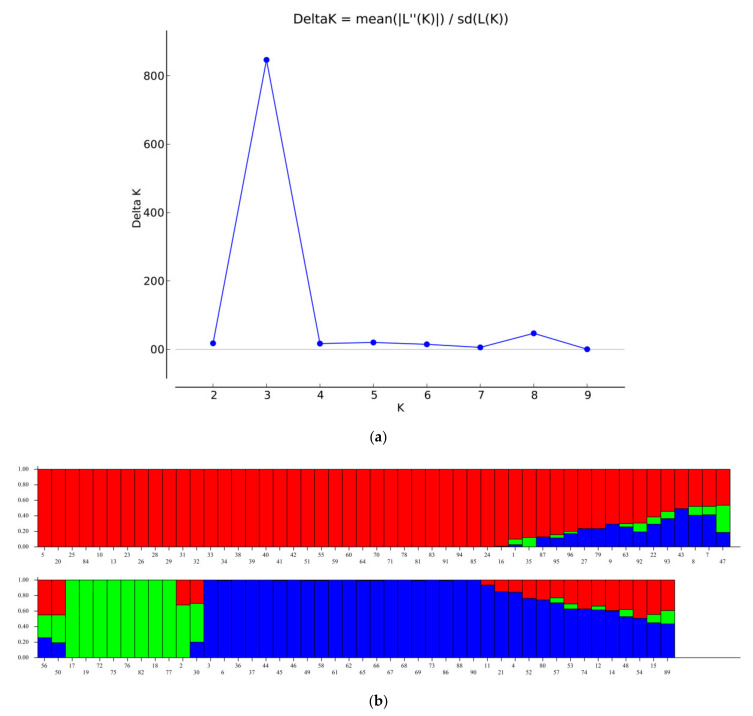
STRUCTURE analysis. (**a**) The ΔK value was highest for the model parameter K = 3 than for other values of K. (**b**) The bar plot showing each rice variety belonging to three subpopulations.

A set of 96 germplasm lines in the current study was subjected to population structure analysis. Based on Evanno plot, ΔK value was highest for the model parameter K = 3 (Figure 4a). Therefore, the optimal number of sub-populations (K) was determined to be 3, which are represented as POP1, POP2, and POP3 (Figure 4b). POP1 was the largest sub-population and constituted of 52 genotypes, of which 38 were pure types and 14 were admixture types. POP2 consisted of 10 genotypes including 8 pure types and 2 admixture types, while POP3 comprised of 34 genotype including 23 pure types and 11 were admixed. The fixation index (Fst) was 0.75, 0.96, and 0.83 for the sub-populations POP1, POP2, and POP3, respectively, and the average distance between individuals within a sub-population was 0.12, 0.03, and 0.09, respectively. The allelic frequency divergence of POP1 from POP2 was 0.49, and from POP3 was 0.25, while between POP2 and POP3 it was 0.45.

### 2.4. Genome-Wide Association Study for Traits Associated with Salinity Tolerance

**Figure 5:**
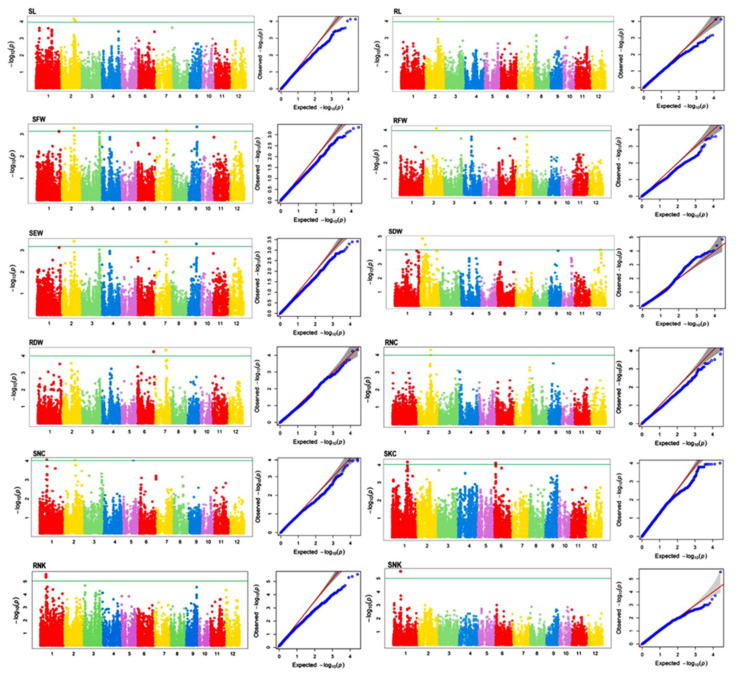
Manhattan plots and Q–Q plots representing the significant marker trait associations for 12 seedling salt tolerance related traits. SL, shoot length (cm); RL, root length (cm); SFW, shoot fresh weight (g); RFW, root fresh weight (g); SEW, seedling weight (g); SDW, shoot dry weight (g); RDW, root dry weight (g); RNC, root Na^+^ content (mmol/g); SNC, shoot Na^+^ content (mmol/g); SKC, shoot K^+^ content (mmol/g); RNK, root Na^+^/K^+^ ratio; SNK, shoot Na^+^/K^+^ ratio.

A total of 23 MTAs were identified for twelve traits in the current study. Out of 23 MTAs, 21 MTAs were located either in or near the previously reported QTLs/MTAs associated with seedling stage salinity tolerance and two MTAs were novel. These MTAs are located on rice chromosomes 1, 2, 5, 6, 7, 9, and 12 and explained the trait phenotypic variance ranging from 13.98% to 29.88% (Table 1). Manhattan plots and quantile–quantile (Q-Q) plots generated through the model are presented in Figure 5. The Q-Q plots indicate that the model was well fitted to the data as the observed *p*-values showed less deviation from the expected *p*-values.

For SL and RL, one common MTA (SNP-AX-95920196) was identified on chromosome 2 and explained the trait phenotypic variance of 15.11% and 19.26%, respectively. For SFW and SEW, three common MTAs (SNP-AX-95921620, SNP-AX-95937657, and SNP-AX-95931839) were identified on chromosome 2, 7, and 9 and explained the phenotypic variance ranging from 13.98% to 15.43 %. One MTA (SNP-AX-95921620) was identified for RFW on chromosome 2 with phenotypic variance 14.64 %. For SDW, a total of three MTAs (SNP-AX-95920663, SNP-AX-95934798, and SNP-AX-95939149) were identified on chromosome 2 and 12 and explained 25.51%, 22.52%, and 20.24% of phenotypic variance, respectively. For RDW, two MTAs (SNP AX-95956901 and SNP AX-95929366) were identified on chromosome 6 and 7 and explained the trait phenotypic variance of 22.05% and 22.62%, respectively.

For RNC, two MTAs (SNP-AX-95921298 and SNP- AX-95920628) were identified on chromosome 2, and explained 18.17% and 15.15% of phenotypic variance. For SNC, three MTAs (SNP- AX-95940587 SNP-AX-95920537, and SNP-AX-95927105) were identified on chromosome 1, 2, and 5 and explained the trait phenotypic variance of 16.26%, 16.64%, and 18.08%, respectively. For SKC, two MTAs (SNP-AX-95940587 and SNP-AX-95937335) were identified on chromosome 1 and 6 and explained 16.24% and 14.33% of phenotypic variance. For RNK and SNK, one MTAs each was identified on chromosome 1 (SNPAX-95918556 and SNP-AX-95940642), explaining the trait phenotypic variance of 25.11 and 29.88%, respectively.

In *silico* search for annotated gene MSU-RAP database led to identification of 18 and 13 putative candidate genes in ~200 Kb genomic region of identified novel MTAs, qSDW2.1 and qSNC5, respectively (Table 2).

## 3. Discussion

Advancement in breeding programs towards development of salt tolerant rice varieties would have a major impact on global food security especially when the world is facing the issue of raising sea level due to global warming [48]. Soil salinity is the critical abiotic stress and a major problem of rice-based farming systems in the coastal areas. Also, inland is affected by salinity due to continuous use of underground brackish water for irrigation. Around 8% of rainfed and 20% of the irrigated agricultural land is affected by salinity [49]. Therefore, development of rice varieties with tolerance to salinity stress is of utmost importance. Huge genetic variability for seedling and reproductive stage salinity tolerance has been reported in rice [4,28,50,51], thus making it acquiescent to genetic manipulation for improving tolerance to salinity stress [52,53]. In the present study, we have evaluated a set of genotypes consisting of landraces, cultivars, and breeding lines for seedling-stage salinity tolerance. We observed the tremendous diversity for all measured traits and identified eleven genotypes of diverse origin that showed seedling stage salinity tolerance. These lines can be used to as donor source of salt tolerance in rice breeding programs and also to understand the mechanism of salinity tolerance.

Morpho-physiological traits related to seedling biomass are affected due to excess salt injury [54]. As observed in this study, the influence of salinity has greater effect on shoot than on root, indicating sensitivity of shoots over roots for salinity stress [55,56]. The decrease in root and shoot lengths might be due to direct or indirect effect of salinity on cell division and expansion [57]. Additionally, harmful effects on photosynthesis due to alterations in enzyme activity may lead to reduction in synthesis of protein, carbohydrates, and growth hormones [56]. The key component of seedling performance under stress is seedling weight. Tolerant genotypes accumulated higher biomass while sensitive genotypes accumulated less biomass. This may be attributed to the genetic capability of tolerant genotypes to retain osmotic potential under salt stress [57], while sensitive genotypes succumbed to ionic toxicity that incited cell turgor loss leading to leaf rolling and stomatal closure consequently disrupting the photosynthesis activity [58].

Under salt stress conditions genotypes which were tolerant (with STS 3) accumulated less Na^+^ and high K^+^ in both root and shoot compared to sensitive genotypes (with STS 7 & 9). Furthermore, significant negative correlation between Na^+^ ion concentration of root and shoot with seedling weight, length, fresh weight, and dry weight of root and shoot was observed. Reduced uptake of sodium while increasing the uptake of potassium is one of the critical salt tolerance mechanisms [17,59,60,61,62]. Under salt stress conditions, due to accumulation of Na^+^, there is significant decrease in chlorophyll concentration which limits the photosynthetic capacity of salt-sensitive plants, leading to chlorosis and reduced growth of seedlings [4,20,63]. This strong association of low Na^+^ uptake, high K^+^ uptake and low Na^+^/K^+^ ratio with salt tolerance was formerly reported in many studies [28,62,64]. The *SKC1* gene from Nona Bokra regulates Na^+^/K^+^ homeostasis in the shoot under salt stress conditions [59]. In the current study, 11 salt tolerant genotypes (UPRI-2003-45, Samanta, Tompha Khau, Chandana, Narendra Usar Dhan II, Narendra Usar Dhan III, PMK-1, Seond Basmati, Manaswini and Shah Pasand) with higher concentration of K^+^ and low Na^+^/K^+^ were identified (Supplementary Table S1) which could be worthy candidates of seedling stage salt tolerance in rice breeding programs.

Identifying the genomic regions governing this complex trait is of utmost importance to develop high yielding salinity tolerant rice varieties. Association mapping takes advantage of historical recombination and mutational events in order to precisely detect MTAs [65]. However, familial relatedness and population structure leads to false positives and false negatives. In the current study, three sub-populations were detected which were considered in mixed linear model (MLM) to reduce spurious associations. Ever since the publication of MLM, it has been popularly adopted for GWAS in crops [66,67,68]. Though, MLM being a single locus method that allows testing of one marker locus at a time, had an intrinsic limitation in matching the real genetic architecture of the complex traits that are under the effect of multiple loci acting simultaneously [69]. Latest studies on plant height and flowering time [70], ear traits [71], and starch pasting properties in maize [71], yield-related features in wheat [72], stem rot resistance in soybean [73], agronomic traits in foxtail millet [74], panicle architecture in sorghum [75], and most recently Fe and Zn content in rice grain [76] have established the power of fixed and random model circulating probability unification (FarmCPU) model that uses both fixed effect and random effect models iteratively to effectively control the false findings. The present study discovered FarmCPU as a best-fit model with better power of test statistics after a comparison of Q–Q plots obtained through different models. The threshold of -log10(P) >3 was used to declare MTAs because of restricted number of genotypes used in the study. In one of the latest studies, Rohilla et al. [77] used 94 deep-water rice genotypes of India in GWAS for anaerobic germination (AG) and found significant associated SNPs at log10(P) =3. Similarly, Biselli et al. [78] conducted GWAS for starch-related parameters in 115 japonica rice and used the threshold of log10(P) = 3. Feng et al. [79] performed GWAS for grain shape traits in indica rice and found significant associated SNPs at log10(P) = 3. Kim and Reinke [80] identified a novel bacterial leaf blight resistant gene *Xa43(t)* at −log10(P) value of 4 which was further validated in a bi-parental mapping population.

In the present study, we have identified a total of 23 MTAs for 12 seedling stage salinity tolerance associated traits. These QTLs are located on rice chromosomes 1, 2, 5, 6, 7, 9, and 12 and explained the trait phenotypic variances ranging from 13.98% to 29.88%. We compared the MTAs identified in this study with previously reported QTLs related to salinity tolerance in the QTL Annotation Rice Online (Q-TARO) database and by literature survey. This comparison showed that 18 MTAs identified in this study were located either in or near the previously reported QTLs associated with seedling stage salinity tolerance (Table 1). The genomic region associated with qSL2 and qRL2 were earlier reported to govern seedling height (qPH2: RM13197-RM6318) [24] and germination percentage (qGP2: RM8254-RM5804) [41] under salt stress. The MTAs, qSFW2, qRFW2, and qSEW2 were detected in the genomic regions reported to govern seedling height and root K^+^ concentration (qPH2, qRKC2: RM13197-RM6318), leaf chlorophyll content (qCHl2: RM12713-RM6318) [24] and germination percentage (qGP2: RM8254-5804) [41] under salt stress. The MTAs qSFW7 were located in the vicinity of genomic region reported to be associated with relative shoot dry weight (qRSW7: RM560) under salt stress [42]. The MTAs qSFW9 were located in the genomic region reported to be associated with shoot and root Na^+^/K^+^ ratio (qSNK9, qRNK9: RM296-RM7175) [24] and germination percentage (qGP9: RM219-RM7048) [41]. The candidate gene, *OsGMST1* (LOC_Os02g17500-10.07 Mb on chromosome 2) located near the MTA qSDW2.2 (AX-95934798-10.21 Mb) is known to induce under salinity stress and govern tolerance [44]. The MTA qSDW12.1 was identified in the genomic region reported to be associated with initial and final standard evaluation system (SES) score, seedling survival and leaf chlorophyll content (qSES12, qSUR12, and qCHL12: RM27933-RM17) [24]. A large amount of truncated proteins derived from the candidate gene *OsCMO* (29.34 Mb on chromosome 6) located near the MTA qRDW6 (AX-95956901-29.72 Mb) was reported to induce in response to salinity stress [45].The MTA qRDW7 was located in the vicinity of genomic region reported to be associated with root K^+^ concentration and stress evaluation score (qSES7.1; qRK7.1: HvSSR 07-25—HvSSR 07-37) at reproductive stage salt stress [46]. The MTAs qRNC2.1 and qRNC2.2 were located in the genomic region reported to be associated with root K^+^ concentration (qRKC2: RM13197-RM6318) [24] and germination percentage in 10 days (qGP2:RM8254-RM5804) [41]. The MTAs qSNC1, qSKC1, and qSNK1 were located in the genomic region reported to be associated with shoot K^+^ concentration (qSKC1:RM8094-RM10825) and shoot and root Na^+^/K^+^ ratio (qSNK1; qRNK1: RM1287-RM1025) [24]. The MTA, qSKC6 (AX-95937335-3.42 Mb) identified under salt stress conditions was located in the genomic region reported to be associated with RDW and RFW under control conditions (qRFWn6.1; qRDWn6.1 at 3.59 Mb) [47]. The MTAs, qRNK1 was positioned in the genomic region reported to accountable for shoot Na^+^ concentration (qSNC1: RM1287-RM10793), shoot and root K^+^ concentration (qSKC1: RM8094-RM10825; qRKC1: RM1287-RM11300) and root Na^+^/K^+^ ratio qRNK1: RM1287-RM10825) [24].

The candidate gene *Os01g0304100* (11.26Mb on chromosome 1) located near the MTA qRNK1 (AX-95918556-11.02 Mb) encode cation chloride co-transporter which was identified as a determinant of salt tolerance in previous works [16]. The candidate gene *OsVTE1* (LOC_Os02g17650-10.15 Mb on chromosome 2) located near the MTA qSDW2.2 (AX-95934798-10.21 Mb), encode a rice tocopherol cyclase ortholog which was induced significantly by abiotic stresses such as high salt, drought, cold, and by the salicylic acid and abscisic acid plant hormones [43].

Apart from MTAs associated with previously reported known QTLs or genes governing salinity tolerance, we detected two novel MTAs namely, qSDW2.1 and qSNC5. In *silico* analysis revealed that the candidate gene LOC_Os02g10590 in the genomic region of MTA qSDW2.1, was annotated as peptidyl-prolyl cis-trans isomerase (*FKBP*-type) proteins (Table 2). *FKBPs* belong to a large ubiquitous family of proteins which are found in every part of the cell and involved in different processes like protein folding to stress response. Around 20 *FKBPs* are encoded by higher plant genomes, half of which are found in chloroplast thylakoid lumen. Most of the *FKBPs* in plants regulate hormone signaling with main role in plant growth and development, stress response and seed germination. The rice *FKBP* family seems to have developed by duplications of *FKBP* genes which could be an approach for improved stress tolerance [81]. The putative candidate genes LOC_Os02g10600 and LOC_Os02g10700 in the genomic regions of MTAs qSDW2.1, were annotated as *OsFBA1* and *OsFBL7* (F-box and FBA domain containing protein and F-box domain and LRR containing protein), respectively (Table 2). F-box proteins are characterized by a conserved F-box motif and these constitute a large family in eukaryotes. F-box protein-encoding genes have been found to be differentially expressed in rice seedlings exposed to salt stress [82].

The putative candidate gene LOC_Os05g33500 in the genomic region of MTAs qSNC5, was annotated as *mTERF* domain containing protein (Table 2). Plant mitochondrial transcription termination factor (*mTERF*) genes play significant role in regulating organelle gene expression. Environmental stimulus experiments revealed differential up or downregulation expression of maize *mTERF* genes in seedlings exposed to light/dark, salts and plant hormones, respectively, suggesting numerous important roles of maize *mTERF* genes in light acclimation and stress-related responses. The transcript levels of the maize *mTERF12* gene, the ortholog of Arabidopsis *mTERF6*, also those of maize *mTERF13* and *mTERF28*, altered after exposure to NaCl, AlCl_3_, or ABA in comparison to the untreated plants [83]. Therefore, rice *mTERF* gene could possibly play role in the rice seedlings in response to salinity stress and have important role in the growth, and development of rice seedling under stress conditions. These novel MTAs identified in the study may play important role in imparting salinity tolerance in rice.

## 4. Material and Methods

### 4.1. Plant Materials

A set of 96 rice genotypes, consisting of landraces, cultivars and breeding lines were evaluated for seedling stage salinity tolerance under hydroponic conditions. The details of the genotypes used in the current study are presented in Supplementary Table S2. FL478 was used as tolerant check and IR29 was used as susceptible check [24].

### 4.2. Evaluation for Seedling Stage Salinity Tolerance

The experiment for evaluation of seedling stage salinity tolerance was set up under hydroponic conditions in the glass house of National Phytotron Facility, ICAR-IARI, New Delhi, India. The temperature was set to 30℃ during day and 22 °C at night with relative humidity of 65–70%. The seeds were surface sterilized with 5% sodium hypochlorite solution for 30 min and rinsed with distilled water several times. Sterilized seeds were placed on germination paper in Petri dishes and incubated for 72 h. Sterile plastic containers with styrofoam sheets were used for screening the genotypes under hydroponics. Styrofoam with 10 × 16 matrix of hole was used, where the bottom of the hole was covered by stitching nylon net to prevent seeds from falling into the nutrient solution. The plastic tray was filled with 15 liters of modified Yoshida nutrient solution [84] and styrofoam sheet was allowed to float on the solution. The components and concentrations of modified Yoshida nutrient solution are presented in Supplementary Table S3.

One healthy pre-germinated seed was placed in each hole of styrofoam sheet with each genotype in a row (10 holes). Each plastic tray could accommodate 14 test genotypes along with sensitive (IR29) and tolerant (FL478) checks. The entire experimental set up consisted of plastic trays with modified Yoshida nutrient solution in completely randomized design (CRD) with two replications and each genotype comprised of ten plants per replication.

Fourteen days after germination, saline solution with 60 milimolar (mM) NaCl (EC of 6.9 dS/m) was added to the tray and after three days, salinity stress was raised to 120 mM (EC of 13.9 dS/m) which was maintained until final phenotypic scoring. The container was refilled with fresh nutrient solution maintaining the required salinity level and pH of 5.0 at every four days interval. On 16th day after first salinization, the genotypes were visually scored using modified standard evaluation system for rice [85].

### 4.3. Measurement of Morpho-Physiological Characters

After final scoring, three plants per genotype were rinsed three times in distilled water and data on seven traits *viz*., SL, SFW, RL, RFW, SEW, SDW, and RDW were recorded. For each plant, the SL was recorded from the base of the plant to the tip of the longest leaf while RL was measured from the base of the plant to the tip of the longest root. Plants were dried in hot air oven at 80 °C for 72 h and SDW and RDW were measured using a high precision digital balance. Dried samples of shoot and root were used for assessment of Na^+^ and K^+^ ion concentration.

### 4.4. Estimation of Na^+^ and K^+^ Ion Concentration

The Na^+^ and K^+^ ion content of the root and shoot samples were determined using flame photometer as described by Yoshida et al. [84]. The oven dried plant material was ground to fine powder. About 100 mg of the ground powder was added into a test tube containing 15 mL of di-acid digestion mixture (HNO_3_ and HClO_4_, 10:3). The mixture was cooled and transferred to a 50 mL volumetric flask and volume was made up to 50 mL using double distilled water. The mixture was shaken gently and filtered with Whatman number 42 filter paper and concentration of Na^+^ and K^+^ ions was estimated using Systronics Type 128 Flame Photometer (Systronics Gujarat, Ahmedabad, India). Three replicates were performed per sample and the average value of the replicate was taken. The concentration of ions was expressed in millimoles per gram of dry weight (mmol/g of dry wt).

### 4.5. Data Analysis

Descriptive statistics and correlations between traits were worked out using R v.3.6.0. Hierarchical cluster analysis [86] was conducted using Ward’s method [87] and clusters were generated based on Euclidean distance using package ‘dendextend’ version 1.13.4 in R version 3.6.3.

### 4.6. DNA Isolation and SNP Genotyping

Total genomic DNA was isolated from young leaves using Cetyl Trimethyl Ammonium Bromide (CTAB) method [88]. The DNA was quantified using a nano-drop spectrophotometer (NanoDrop^TM^ 2000/2000 c, Thermo Fisher Scientific, DE, United States). High throughput genotyping was carried out using a *“OsSNPnks”* 50 K genic Affymetrix chip containing a total of 50,051 high-quality SNPs. The chip was based on single copy genes, covering all 12 rice chromosomes [89]. DNA amplification, fragmentation, chip hybridization, single-base extension through DNA ligation, and signal amplification was carried out using the method suggested by Singh et al. [89].

### 4.7. Population Structure Analysis

The software STRUCTURE 2.3.4 [90] was utilized to determine the population structure of 96 genotypes using a Bayesian model of ADMIXTURE [91], wherein 10 independent runs with 50,000 burn-ins and Markov Chain Monte Carlo (MCMC) period set to 50,000 was conducted for each K. Furthermore, STRUCTURE HARVESTER [92] was used to estimate the optimum number of sub-populations [93].

### 4.8. Genome-Wide Association Study

Association mapping panel of 96 germplasm lines were genotyped using 50 K SNP chip. SNP data was filtered for minor allele frequency (MAF) ≥0.05 and maximum missing sites per SNP was fixed to <20%. After filtering, a total of 26,108 SNPs were used to detect MTAs. MTAs were identified using MLM and FarmCPU [94,95] implemented in GAPIT (genomic association and prediction integrated tool).

## 5. Conclusions

Morphological and physiological characterization of rice germplasm lines identified 11 genotypes tolerant to salinity stress. These lines can be utilized as donors in a breeding program for developing salt tolerant rice varieties. Furthermore, GWAS identified a total of 23 MTAs for traits associated with seedling stage salinity tolerance, of which two are novel.

## Figures and Tables

**Table 1:** Details of the marker-trait associations (MTAs) identified for salt tolerance related traits at seedling stage.

S.No	Traits	MTAs	SNP	Chr.	Position	*p* Value	R^2^	Previous Report
1	SL	qSL2	AX-95920196	2	23947447	6.74 × 10^−5^	15.11	qPH2 [24]; qGP-2 [41]
2	RL	qRL2	AX-95920196	2	23947447	7.54 × 10^−5^	19.26	qPH2 [24]; qGP-2 [41]
3	SFW	qSFW2	AX-95921620	2	23533590	5.18 × 10^−4^	14.64	qPH2, qRKC2, qCHL2 [24]; qGP-2 [41]
		qSFW7	AX-95937657	7	20788892	6.79 × 10^−4^	13.98	qRSW7 [42]
		qSFW9	AX-95931839	9	16483542	4.61 × 10^−4^	14.92	qSNK9, qRNK9, qSES9 [24]; qGP-9 [41]
4	RFW	qRFW2	AX-95921620	2	23533590	5.18 × 10^−4^	14.64	qPH2, qRKC2, qCHL2 [24]; qGP-2 [41]
5	SEW	qSEW2	AX-95921620	2	23533590	3.87 × 10^−4^	15.43	qPH2, qRKC2, qCHL2 [24]; qGP-2 [41]
		qSEW7	AX-95937657	7	20788892	4.07 × 10^−4^	15.31	qRSW7 [42]
		qSEW9	AX-95931839	9	16483542	5.06 × 10^−4^	14.77	qSNK9, qRNK9 [24]; qGP-9 [41]
6	SDW	qSDW2.1	AX-95920663	2	5664763	1.42 × 10^−5^	25.51	-
		qSDW2.2	AX-95934798	2	10213902	4.05 × 10^−5^	22.52	*OsVTE1* [43]; *OsGMST1* [44]
		qSDW12.1	AX-95939149	12	17404747	9.22 × 10^−5^	20.24	qSES12, qSUR12, qCHL12 [24]
7	RDW	qRDW6	AX-95956901	6	29729562	5.79 × 10^−5^	22.05	*OsCMO* [45]
		qRDW7	AX-95929366	7	20782724	4.74 × 10^−5^	22.62	qSES7.1, KR7.1 [46]
8	RNC	qRNC2.1	AX-95921298	2	23260124	6.30 × 10^−5^	18.17	qRKC2 [24]; qGP-2 [41]
		qRNC2.2	AX-95920628	2	22172032	9.45 × 10^−5^	15.15	qRKC2 [24]; qGP-2 [41]
9	SNC	qSNC1	AX-95940587	1	13758487	8.16 × 10^−5^	16.26	qSKC, qSNK, qRNK [24]
		qSNC2	AX-95920537	2	22171212	8.35 × 10^−5^	16.64	qRKC2 [24]; qGP-2 [41]
		qSNC5	AX-95927105	5	19697164	9.39 × 10^−5^	18.08	-
10	SKC	qSKC1	AX-95940587	1	13758487	7.86 × 10^−5^	16.26	qSKC, qSNK, qRNK [24]
		qSKC6	AX-95937335	6	3429601	8.21 × 10^−5^	14.33	qRFWn6.1, qRDWn6.1 [47]
11	RNK	qRNK1	AX-95918556	1	11022718	6.26 × 10^−6^	25.11	qRNK1, qSNC, qSKC, qRKC [24]
12	SNK	qSNK1	AX-95940642	1	13322813	2.93 × 10^−6^	29.88	qSKC, qSNK, qRNK [24]

**Table 2:** Putative candidate genes annotated in ~200 Kb genomic regions in identified novel MTAs qSDW2.1 and qSNC5.

Chr.	MSU-RAP ID	Position (bp)	Description or Putative Function
2	LOC_Os02g10580	5,565,956	NB-ARC domain containing disease resistance protein
	LOC_Os02g10590	5,569,801	Peptidyl-prolyl cis-trans isomerase, *FKBP*-type
	LOC_Os02g10600	5,573,182	*OsFBA1*—F-box and FBA domain containing protein
	LOC_Os02g10630	5,593,645	*GRAM* and C2 domains containing protein
	LOC_Os02g10640	5,600,889	26S protease regulatory subunit,
	LOC_Os02g10650	5,604,236	*CRAL/TRIO* domain containing protein
	LOC_Os02g10660	5,614,461	Gycosyl hydrolases family 17
	LOC_Os02g10690	5,623,352	Targeting protein for *Xklp2*
	LOC_Os02g10700	5,631,315	*OsFBL7*—F-box domain and LRR containing protein
	LOC_Os02g10710	5,640,360	hsp20/alpha crystallin family protein
	LOC_Os02g10750	5,672,334	CBL-interacting protein kinase
	LOC_Os02g10760	5,688,698	*AP2* domain containing protein
	LOC_Os02g10770	5,697,834	*DEAD*-box ATP-dependent RNA helicase 41
	LOC_Os02g10780	5,706,636	*SPX* domain containing protein
	LOC_Os02g10800	5,736,606	Mitochondrial carrier protein
	LOC_Os02g10810	5,742,520	Protein of unknown function domain containing protein
	LOC_Os02g10820	5,748,141	*Sel1* repeat domain containing protein
	LOC_Os02g10830	5,749,500	Serine acetyltransferase protein
5	LOC_Os05g33500	19,678,142	*mTERF* domain containing protein
	LOC_Os05g33510	19,681,732	Peptide methionine sulfoxide reductase *msrB*
	LOC_Os05g33550	19,704,966	Methyl-binding domain protein MBD
	LOC_Os05g33554	19,707,432	Methyl-binding domain protein MBD
	LOC_Os05g33570	19,737,857	Pyruvate, phosphate dikinase, chloroplast precursor
	LOC_Os05g33590	19,744,851	Cytochrome P450, putative, expressed
	LOC_Os05g33600	19,758,913	Cytochrome P450 72A1, putative, expressed
	LOC_Os05g33630	19,785,962	Inosine-uridine preferring nucleoside hydrolase family protein
	LOC_Os05g33644	19,805,637	Inosine-uridine preferring nucleoside hydrolase family protein
	LOC_Os05g33690	19,828,422	Receptor-like protein kinase precursor
	LOC_Os05g33700	19,834,008	4F5 protein family protein
	LOC_Os05g33710	19,846,592	WD domain, G-beta repeat domain containing protein
	LOC_Os05g33730	19,868,419	Gibberellin receptor *GID1L2*

## Data Availability

Data used in this study are presented in the Supplementary Materials.

## Supplementary Materials

The following are available online at https://www.mdpi.com/2223-7747/10/3/559/s1, Figure S1: Correlation between the traits for seedling stage salinity tolerant. Table S1: Morpho-physiological traits and biochemical data of the rice genotypes evaluated for seedling stage salinity tolerance. Table S2: Details of germplasm used in the current study; Table S3: The chemical composition of modified Yoshida nutrient solution used in the study.

## References

[1] FAOSTAT (2017). FAO Rice Market Monitor.

[2] Turral H., Burke J., Faurès J.M. (2011). Climate Change, Water and Food Security.

[3] Bouman B.A., Barker R., Humphreys E., Tuong T.P., Atlin G., Bennett J., Dawe D., Dittert K., Dobermann A., Facon T. (2007). Rice: Feeding the Billions. Water for Food, Water for Life: A Comprehensive Assessment of Water Management in Agriculture.

[4] Munns R., Tester M. (2008). Mechanisms of salinity tolerance. Annu. Rev. Plant Biol..

[5] Provin T., Pitt J.L. (2001). Managing Soil Salinity. https://oaktrust.library.tamu.edu/bitstream/handle/1969.1/86985/pdf_1397.pdf?sequence=1.

[6] Lutts S., Kinet J.M., Bouharmont J. (1995). Changes in plant response to NaCl during develop-ment of rice (*Oryza sativa L*.) varieties differing in salinity resistance. J. Exp. Bot..

[7] García A., Rizzo C., Ud-Din J., Bartos S., Senadhira D., Flowers T., Yeo A. (1997). Sodium and potassium transport to the xylem are inherited independently in rice, and the mechanism of sodium: Potassium selectivity differs between rice and wheat. Plant Cell Environ..

[8] Zeng L., Shannon M.C. (2000). Salinity Effects on Seedling Growth and Yield Components of Rice. Crop. Sci..

[9] Tester M., Davenport R. (2003). Na+ tolerance and Na+ transport in higher plants. Ann. Bot..

[10] Horie T., Karahara I., Katsuhara M. (2012). Salinity tolerance mechanisms in glycophytes: An overview with the central focus on rice plants. Rice.

[11] Todaka D., Nakashima K., Shinozaki K., Yamaguchi-Shinozaki K. (2012). Toward understand-ing transcriptional regulatory networks in abiotic stress responses and tolerance in rice. Rice.

[12] Grieve C.M., Grattan S.R., Maas E.V. (2012). Plant salt tolerance. ASCE Man. Rep. Eng. Pract..

[13] Umali D.L. (1993). Irrigation-Induced Salinity: A Growing Problem for Development and the Environment.

[14] Gregoria G.B., Senadhira D., Mendoza R.D. (1997). Screening Rice for Salinity Tolerance. http://www.knowledgebank.irri.org/ricebreedingcourse/documents/Screening_manual.pdf.

[15] Gregorio G.B., Senadhira D. (1993). Genetic analysis of salinity tolerance in rice (*Oryza sativa L*.). Theor. Appl. Genet..

[16] Walia H., Wilson C., Condamine P., Liu X., Ismail A.M., Zeng L., Wanamaker S.I., Mandal J., Xu J., Cui X. (2005). Comparative transcriptional profiling of two con-trasting rice genotypes under salinity stress during the vegetative growth stage. Plant Physiol..

[17] Bonilla P., Dvorak J., Mackell D., Deal K., Gregorio G. (2002). RFLP and SSLP Mapping of Salinity Tolerance Genes in Chromosome 1 of Rice (Oryza sativa L.) Using Recombinant Inbred Lines. Philippine Agricultural Scientist (Philippines).

[18] Lee K.-S., Choi W.-Y., Ko J.-C., Kim T.-S., Gregorio G.B. (2003). Salinity tolerance of japonica and indica rice (*Oryza sativa L*.) at the seedling stage. Planta.

[19] Koyama M.L., Levesley A., Koebner R.M., Flowers T.J., Yeo A.R. (2001). Quantitative Trait Loci for Component Physiological Traits Determining Salt Tolerance in Rice. Plant Physiol..

[20] Lin H.X., Zhu M.Z., Yano M., Gao J.P., Liang Z.W., Su W.A., Hu X.H., Ren Z.H., Chao D.Y. (2004). QTLs for Na^+^ and K^+^ uptake of the shoots and roots controlling rice salt tolerance. Theor. Appl. Genet..

[21] Yao M.Z., Wang J.F., Chen H.U., Zhai H.Q., Zhang H.S. (2005). Inheritance and QTL map-ping of salt tolerance in rice. Rice Sci..

[22] Lee S.Y., Ahn J.H., Cha Y.S., Yun D.W., Lee M.C., Ko J.C., Lee K.S., Eun M.Y. (2007). Mapping QTLs related to salinity tolerance of rice at the young seedling stage. Plant Breed..

[23] Sabouri H., Sabouri A. (2008). New evidence of QTLs attributed to salinity tolerance in rice. Afr. J. Biotechnol..

[24] Thomson M.J., de Ocampo M., Egdane J., Rahman M.A., Sajise A.G., Adorada D.L., Tumimbang-Raiz E., Blumwald E., Seraj Z.I., Singh R.K. (2010). Charac-terizing the Saltol quantitative trait locus for salinity tolerance in rice. Rice.

[25] Islam M.R., Hassan L., Salam M.A., Collard B.C., Singh R.K., Gregorio G.B. (2011). QTL mapping for salinity tolerance at seedling stage in rice. Emir. J. Food Agric..

[26] Cheng L., Wang Y., Meng L., Hu X., Cui Y., Sun Y., Zhu L., Ali J., Xu J., Li Z. (2012). Identification of salt-tolerant QTLs with strong genetic background effect using two sets of reciprocal introgression lines in rice. Genome.

[27] Chen T., Zhu Y., Chen K., Shen C., Zhao X., Shabala S., Shabala L., Meinke H., Venkataraman G., Chen Z. (2020). Identification of new QTL for salt tolerance from rice variety Pokkali. J. Agron. Crop. Sci..

[28] Yadav A.K., Kumar A., Grover N., Ellur R.K., Krishnan S.G., Bollinedi H., Bhowmick P.K., Vinod K.K., Nagarajan M., Krishnamurthy S.L. (2020). Marker aided introgression of ‘Saltol’, a major QTL for seedling stage salinity tolerance into an elite Basmati rice variety ‘Pusa Basmati 1509’. Sci. Rep..

[29] Cardon L.R., Bell J.I. (2001). Association study designs for complex diseases. Nat. Rev. Genet..

[30] Wang J., McClean P.E., Lee R., Goos R.J., Helms T. (2008). Association mapping of iron deficiency chlorosis loci in soybean (Glycine max L. Merr.) advanced breeding lines. Theor. Appl. Genet..

[31] Mackay I., Powell W. (2007). Methods for linkage disequilibrium mapping in crops. Trends Plant Sci..

[32] Zhu C., Gore M., Buckler E.S., Yu J. (2008). Status and Prospects of Association Mapping in Plants. Plant Genome.

[33] Yu J., Buckler E.S. (2006). Genetic association mapping and genome organization of maize. Curr. Opin. Biotechnol..

[34] Myles S., Peiffer J., Brown P.J., Ersoz E.S., Zhang Z., Costich D.E., Buckler E.S. (2009). As-sociation mapping: Critical considerations shift from genotyping to experimental design. Plant Cell.

[35] Negrão S., Courtois B., Ahmadi N., Abreu I., Saibo N., Oliveira M. (2011). Recent Updates on Salinity Stress in Rice: From Physiological to Molecular Responses. Crit. Rev. Plant Sci..

[36] Shi Y., Gao L., Wu Z., Zhang X., Wang M., Zhang C., Zhang F., Zhou Y., Li Z. (2017). Ge-nome-wide association study of salt tolerance at the seed germination stage in rice. BMC Plant Biol..

[37] Patishtan J., Hartley T.N., de Carvalho R.F., Maathuis F.J. (2018). Genome-wide associa-tion studies to identify rice salt-tolerance markers. Plant Cell Environ..

[38] Lekklar C., Pongpanich M., Suriya-Arunroj D., Chinpongpanich A., Tsai H., Comai L., Chadchawan S., Buaboocha T. (2019). Genome-wide association study for salinity tolerance at the flowering stage in a panel of rice accessions from Thailand. BMC Genom..

[39] Naveed S.A., Zhang F., Zhang J., Zheng T.-Q., Meng L.-J., Pang Y.-L., Xu J.-L., Li Z.-K. (2018). Identification of QTN and candidate genes for Salinity Tolerance at the Germination and Seedling Stages in Rice by Genome-Wide Association Analyses. Sci. Rep..

[40] An H., Liu K., Wang B., Tian Y., Ge Y., Zhang Y., Tang W., Chen G., Yu J., Wu W. (2020). Genome-wide association study identifies QTLs conferring salt tolerance in rice. Plant Breed..

[41] Wang Z., Wang J., Bao Y., Wu Y., Zhang H. (2010). Quantitative trait loci controlling rice seed germination under salt stress. Euphytica.

[42] Tian L., Tan L., Liu F., Cai H., Sun C. (2011). Identification of quantitative trait loci associated with salt tolerance at seedling stage from Oryza rufipogon. J. Genet. Genom..

[43] Ouyang S., He S., Liu P., Zhang W., Zhang J., Chen S. (2011). The role of tocopherol cyclase in salt stress tolerance of rice (Oryza sativa). Sci. China Life Sci..

[44] Cao H., Guo S., Xu Y., Jiang K., Jones A.M., Chong K. (2011). Reduced expression of a gene encoding a Golgi localized monosaccharide transporter (OsGMST1) confers hypersensitivity to salt in rice (*Oryza sativa L*.). J. Exp. Bot..

[45] Luo D., Niu X., Yu J., Yan J., Gou X., Lu B.-R., Liu Y. (2012). Rice choline monooxygenase (*OsCMO*) protein functions in enhancing glycine betaine biosynthesis in transgenic tobacco but does not accumulate in rice (*Oryza sativa L*. ssp. japonica). Plant Cell Rep..

[46] Pundir P., Devi A., Krishnamurthy S.L., Sharma P.C., Vinaykumar N.M. (2021). QTLs in salt rice variety CSR10 reveals salinity tolerance at reproductive stage. Acta Physiol. Plant..

[47] Nayyeripasand L., Garoosi G.A., Ahmadikhah A. (2021). Genome-wide association study (GWAS) to identify salt-tolerance QTLs carrying novel candidate genes in rice during early vegetative stage. Rice.

[48] Jahan N., Zhang Y., Lv Y., Song M., Zhao C., Hu H., Cui Y., Wang Z., Yang S., Zhang A. (2019). QTL analysis for rice salinity tolerance and fine mapping of a candidate locus qSL7 for shoot length under salt stress. Plant Growth Regul..

[49] Asif M.A., Schilling R.K., Tilbrook J., Brien C., Dowling K., Rabie H., Short L., Trittermann C., Garcia A., Barrett-Lennard E.G. (2018). Mapping of novel salt tolerance QTL in an Excalibur × Kukri doubled haploid wheat population. Theor. Appl. Genet..

[50] Ismail A.M., Heuer S., Thomson M.J., Wissuwa M. (2007). Genetic and genomic approaches to develop rice germplasm for problem soils. Plant Mol. Biol..

[51] Singh R.K., Flowers T.J., Pessarakli M. (2010). The Physiology and Molecular Biology of the Effects of Salinity on Rice. Handbook of Plant and Crop Stress.

[52] Akbar M., Yabuno T., Nakao S. (1972). Breeding for Saline-resistant Varieties of Rice: I. Variabil-ity for Salt Tolerance among Some Rice Varietles. Jpn. J. Breed..

[53] Flowers T.J., Yeo A.R. (1981). Variability in the resistance of sodium chloride salinity within rice (*Oryza sativa* L.) varieties. New Phytol..

[54] Misra A.N., Sahu S., Misra M., Singh P., Meera I., Das N., Kar M., Sahu P. (1997). Sodium chloride induced changes in leaf growth, and pigment and protein contents in two rice cultivars. Biol. Plant..

[55] An P., Inanaga S., Li X.J., Eneji A.E., Zhu N.W. (2005). Interactive Effects of Salinity and Air Humidity on Two Tomato Cultivars Differing in Salt Tolerance. J. Plant Nutr..

[56] Mazher A.M.A., El-Quesni E.M.F., Farahat M.M. (2007). Responses of ornamental and woody trees to salinity. World J. Agric. Sci..

[57] Munns R. (2002). Comparative physiology of salt and water stress. Plant Cell Environ..

[58] Gale J., Zeroni M. (1984). Cultivation of Plants in Brackish Water in Controlled Environment Agriculture. https://agris.fao.org/agris-search/search.do?recordID=US19860060131.

[59] Ren Z.H., Gao J.P., Li L.G., Cai X.L., Huang W., Chao D.Y., Zhu M.Z., Wang Z.Y., Luan S., Lin H.X. (2005). A rice quantitative trait locus for salt tolerance encodes a sodium trans-porter. Nat. Genet..

[60] Pushparajan N., Krishnasamy V., Babu R.C., Kannanbabu J.R. (2011). Association mapping of salinity tolerance in rice using molecular markers. Int. J. Biol. Stress Manag..

[61] Wang Z., Chen Z., Cheng J., Lai Y., Wang J., Bao Y., Huang J., Zhang H. (2012). QTL Analysis of Na+ and K+ Concentrations in Roots and Shoots under Different Levels of NaCl Stress in Rice (*Oryza sativa L*.). PLoS ONE.

[62] De Leon T.B., Linscombe S., Gregorio G., Subudhi P.K. (2015). Genetic variation in Southern USA rice genotypes for seedling salinity tolerance. Front. Plant Sci..

[63] Apse M.P., Aharon G.S., Blumwald E. (1999). Salt Tolerance Conferred by Overexpression of a Vacuolar Na+/H+ Antiport in Arabidopsis. Science.

[64] Babu N.N., Vinod K.K., Krishnamurthy S.L., Krishnan S.G., Yadav A., Bhowmick P.K., Nagarajan M., Singh N.K., Prabhu K.V., Singh A.K. (2016). Microsatellite based linkage disequilibrium analyses reveal Saltol haplotype fragmentation and identify novel QTLs for seedling stage salinity tolerance in rice (*Oryza sativa L*.). J. Plant Biochem. Biotechnol..

[65] Zhao K., Tung C.-W., Eizenga G.C., Wright M.H., Ali M.L., Price A.H., Norton G.J., Islam M.R., Reynolds A.R., Mezey J.G. (2011). Genome-wide association mapping reveals a rich genetic architecture of complex traits in Oryza sativa. Nat. Commun..

[66] Zhang P., Liu X., Tong H., Lu Y., Li J. (2014). Association Mapping for Important Agronomic Traits in Core Collection of Rice (*Oryza sativa L*.) with SSR Markers. PLoS ONE.

[67] Ya-Fang Z., Yu-Yin M., Zong-Xiang C., Jie Z., Tian-Xiao C., Qian-Qian L., Xue-Biao P., Shi-Min Z. (2015). Genome-Wide Association Studies Reveal New Genetic Targets for Five Panicle Traits of International Rice Varieties. Rice Sci..

[68] Wang Y., Zheng Y., Cai Q., Liao C., Mao X., Xie H., Zhu Y., Lian L., Luo X., Xie H. (2016). Population structure and association analysis of yield and grain quality traits in hybrid rice primal parental lines. Euphytica.

[69] Kaler A.S., Purcell L.C. (2019). Estimation of a significance threshold for genome-wide association studies. BMC Genom..

[70] Wallace J.G., Zhang X., Beyene Y., Semagn K., Olsen M., Prasanna B.M., Buckler E.S. (2016). Genome-wide Association for Plant Height and Flowering Time across 15 Tropical Maize Populations under Managed Drought Stress and Well-Watered Conditions in Sub-Saharan Africa. Crop. Sci..

[71] Xu Y., Yang T., Zhou Y., Yin S., Li P., Liu J., Xu S., Yang Z., Xu C. (2018). Genome-Wide Association Mapping of Starch Pasting Properties in Maize Using Single-Locus and Multi-Locus Models. Front. Plant Sci..

[72] Ward B.P., Brown-Guedira G., Kolb F.L., Van Sanford D.A., Tyagi P., Sneller C.H., Griffey C.A. (2019). Genome-wide association studies for yield-related traits in soft red winter wheat grown in Virginia. PLoS ONE.

[73] Wei W., Mesquita A.C.O., Figueiró A.D.A., Wu X., Manjunatha S., Wickland D.P., Hudson M.E., Juliatti F.C., Clough S.J. (2017). Genome-wide association mapping of resistance to a Brazilian isolate of Sclerotinia sclerotiorum in soybean genotypes mostly from Brazil. BMC Genom..

[74] Jaiswal V., Bandyopadhyay T., Gahlaut V., Gupta S., Dhaka A., Ramchiary N., Prasad M. (2019). Genome-wide association study (GWAS) delineates genomic loci for ten nutritional elements in foxtail millet (Setaria italica L.). J. Cereal Sci..

[75] Zhou Y., Srinivasan S., Mirnezami S.V., Kusmec A., Fu Q., Attigala L., Fernandez M.G.S., Ganapathysubramanian B., Schnable P.S. (2019). Semiautomated Feature Extraction from RGB Images for Sorghum Panicle Architecture GWAS. Plant Physiol..

[76] Bollinedi H., Yadav A.K., Vinod K.K., Krishnan S.G., Bhowmick P.K., Nagarajan M., Neeraja C.N., Ellur R.K., Singh A.K. (2020). Genome-Wide Association Study Reveals Novel Marker-Trait Associations (MTAs) Governing the Localization of Fe and Zn in the Rice Grain. Front. Genet..

[77] Rohilla M., Singh N., Mazumder A., Sen P., Roy P., Chowdhury D., Singh N.K., Mondal T.K. (2020). Genome-wide association studies using 50 K rice genic SNP chip unveil genetic architecture for anaerobic germination of deep-water rice population of Assam, India. Mol. Genet. Genom..

[78] Biselli C., Volante A., Desiderio F., Tondelli A., Gianinetti A., Finocchiaro F., Taddei F., Gazza L., Sgrulletta D., Cattivelli L. (2019). GWAS for Starch-Related Parameters in Japonica Rice (*Oryza sativa L*.). Plants.

[79] Feng Y., Lu Q., Zhai R., Zhang M., Xu Q., Yang Y., Wang S., Yuan X., Yu H., Wang Y. (2016). Genome wide association mapping for grain shape traits in indica rice. Planta.

[80] Kim S.-M., Reinke R.F. (2019). A novel resistance gene for bacterial blight in rice, *Xa43*(t) identified by GWAS, confirmed by QTL mapping using a bi-parental population. PLoS ONE.

[81] Gollan P.J., Bhave M. (2009). Genome-wide analysis of genes encoding FK506-binding proteins in rice. Plant Mol. Biol..

[82] Jain M., Nijhawan A., Arora R., Agarwal P., Ray S., Sharma P., Kapoor S., Tyagi A.K., Khurana J.P. (2007). F-box proteins in rice. Genome-wide analysis, classification, tem-poral and spatial gene expression during panicle and seed development, and regulation by light and abiotic stress. Plant Physiol..

[83] Zhao Y., Cai M., Zhang X., Li Y., Zhang J., Zhao H., Kong F., Zheng Y., Qiu F. (2014). Genome-Wide Identification, Evolution and Expression Analysis of *mTERF* Gene Family in Maize. PLoS ONE.

[84] Yoshida S. (1997). Routine Procedure for Growing Rice Plants in Culture Solution.

[85] IRRI (2013). Standard Evaluation System (SES) for Rice.

[86] Johnson S.C. (1967). Hierarchical clustering schemes. Psychometrika.

[87] Ward J.H. (1963). Hierarchical grouping to optimize an objective function. J. Am. Stat. Assoc..

[88] Doyle J.J., Doyle J.L. (1990). Isolation of plant DNA from fresh tissue. Focus.

[89] Singh N., Jayaswal P.K., Panda K., Mandal P., Kumar V., Singh B., Mishra S., Singh Y., Singh R., Rai V. (2015). Single-copy gene based 50 K SNP chip for genetic studies and molecular breeding in rice. Sci. Rep..

[90] Pritchard J.K., Stephens M., Donnelly P. (2000). Inference of population structure using multi-locus genotype data. Genetics.

[91] Alexander D.H., Novembre J., Lange K. (2009). Fast model-based estimation of ancestry in unrelated individuals. Genome Res..

[92] Earl D.A., Vonholdt B.M. (2011). STRUCTURE HARVESTER: A website and program for visualizing STRUCTURE output and implementing the Evanno method. Conserv. Genet. Resour..

[93] Evanno G., Regnaut S., Goudet J. (2005). Detecting the number of clusters of individuals using the software structure: A simulation study. Mol. Ecol..

[94] Liu X.L. (2015). Development of an Iterative Usage of Fixed Effect and Random Effect Models for Powerful and Efficient Genome-Wide Association Studies. Master’s Thesis.

[95] Liu X., Huang M., Fan B., Buckler E.S., Zhang Z. (2016). Iterative Usage of Fixed and Random Effect Models for Powerful and Efficient Genome-Wide Association Studies. PLoS Genet..

