# Supplementary material for: Genome-Wide Association Study Reveals Marker–Trait Associations for Early Vegetative Stage Salinity Tolerance in Rice
Source: Plants (Basel). 2021 Mar 16;10(3):559. doi: 10.3390/plants10030559 (PMC8000697; doi:10.3390/plants10030559)
Supplement: Supplementary file 1 [file plants-10-00559-s001.pdf]

Table S1. Morpho-physiological traits and biochemical data of the rice genotypes evaluated for seedling stage salinity tolerance.

No.	Genotype	STS	SL	RL	SFW	RFW	SEW	SDW	RDW	RNC	SNC	RKC	SKC	RNK	SNK
1	FL 478	3	66.80	18.35	7.75	0.78	8.53	0.45	0.08	0.68	0.56	1.65	1.89	0.41	0.30
2	CR 2461-9	5	51.86	14.00	3.70	0.43	4.12	0.35	0.05	0.96	0.77	1.36	1.41	0.71	0.57
3	UPRI-2003-45	3	58.35	17.65	7.93	0.76	8.69	0.49	0.06	0.70	0.58	1.24	1.76	0.56	0.33
4	PNR 381	7	32.75	12.05	2.09	0.21	2.30	0.19	0.01	1.19	2.01	0.60	0.54	2.02	3.77
5	Pusa Sugandh 3	9	35.25	11.98	1.64	0.20	1.84	0.11	0.02	1.61	1.82	0.35	0.51	4.64	3.61
6	RNRM 7	7	27.65	11.93	1.83	0.15	1.97	0.13	0.02	1.42	1.34	0.38	0.57	3.79	2.35
7	CSR 27	3	66.79	19.80	8.85	0.32	9.17	0.54	0.05	0.77	0.62	1.23	1.85	0.63	0.33
8	Basmati 370	9	30.83	11.68	2.40	0.49	2.88	0.14	0.03	2.01	1.94	0.67	0.37	3.02	5.32
9	NDR-8015-1	9	32.10	12.85	1.12	0.16	1.27	0.15	0.02	0.95	2.08	0.51	0.50	1.86	4.16
10	Samanta	3	51.65	18.00	6.65	0.58	7.23	0.48	0.06	0.74	0.57	1.82	1.80	0.41	0.31
11	Tapaswani	7	13.75	8.05	0.07	0.07	0.14	0.03	0.02	1.28	1.90	0.63	0.66	2.02	1.87
12	Birupa	9	23.00	9.00	0.79	0.09	0.87	0.04	0.02	1.31	2.50	0.99	1.29	0.86	1.19
13	Bhubana	5	42.83	16.65	5.10	0.55	5.64	0.48	0.05	0.85	0.66	1.28	1.70	0.67	0.39
14	Pusa Basmati 1121	9	34.75	11.03	0.96	0.10	1.06	0.06	0.02	1.07	1.80	0.58	0.63	1.86	2.86
15	Tompha khau	3	62.25	24.20	8.06	0.85	8.78	0.56	0.08	0.76	0.76	1.38	1.89	0.55	0.40
16	Chandana	3	46.99	19.93	4.64	0.50	5.14	0.51	0.05	0.53	0.64	1.31	1.80	0.50	0.35
17	VLT-6	3	49.88	16.03	9.25	0.31	5.44	0.44	0.05	0.64	0.50	1.18	1.60	0.54	0.31
18	VOH-PCR-3110	7	32.60	11.50	0.85	0.17	1.02	0.06	0.03	1.20	1.36	0.44	0.84	2.72	1.62
19	BJ-1	9	25.90	11.80	1.62	0.14	1.76	0.07	0.02	2.40	1.89	0.60	0.45	3.99	4.19
20	Kamlesh	9	26.15	13.23	0.61	0.12	0.73	0.09	0.02	1.57	1.70	0.46	0.65	3.44	2.64
21	Narendra Usar Dhan III	3	58.15	20.96	5.90	0.64	6.54	0.50	0.05	0.75	0.65	1.58	1.90	0.47	0.34
22	Narendra Usar Dhan II	3	65.49	23.69	7.19	0.73	7.92	0.56	0.04	0.80	0.57	1.45	1.80	0.55	0.32
23	WGL-14	9	24.93	10.50	0.89	0.08	0.96	0.07	0.02	2.39	1.93	1.44	0.29	1.67	6.67
24	Khara Munga	9	27.25	9.38	0.84	0.18	1.01	0.14	0.02	1.09	2.37	0.69	0.49	1.59	4.83
25	Apo	9	41.88	12.50	1.18	0.17	1.35	0.18	0.02	1.56	1.91	0.55	0.34	2.85	5.65

STS,salt tolerance score; SL, shoot length (cm); RL, root length (cm); SFW, shoot fresh weight (g); RFW, root fresh weight (g); SEW, seedling weight (g); SDW, shoot dry weight (g); RDW, root dry weight (g); RNC, root Na^+ content (mmol/g); SNC, shoot Na^+ content (mmol/g); RKC, root K^+ content (mmol/g); SKC, shoot K^+ content (mmol/g); RNK, root Na^+/K^+ ratio; SNK, shoot Na^+/K^+ ratio.

Table S1. Con.td

No.	Genotype	STS	SL	RL	SFW	RFW	SEW	SDW	RDW	RNC	SNC	RKC	SKC	RNK	SNK
26	CSR 23	3	58.03	18.78	6.49	0.57	7.06	0.52	0.09	0.72	0.66	1.22	1.94	0.59	0.34
27	Pusa 1342	7	17.00	8.13	0.25	0.09	0.34	0.02	0.02	2.03	1.41	0.44	1.09	4.61	1.30
28	Pant Dhan 10	7	24.80	8.88	0.41	0.10	0.50	0.04	0.02	1.31	1.88	0.46	1.00	2.87	1.88
29	UPRI-2003-18	5	49.04	14.85	4.44	0.17	4.61	0.31	0.04	1.14	0.74	1.50	1.48	0.76	0.50
30	UPRI-2003-24	9	23.88	11.63	0.62	0.15	0.77	0.07	0.02	2.67	1.86	0.41	0.37	6.52	5.03
31	Sarjoo 52	5	40.33	13.00	3.83	0.18	4.01	0.25	0.03	1.31	0.77	1.57	1.35	0.84	0.57
32	Nagina 12	7	31.68	11.45	1.15	0.08	1.22	0.06	0.02	2.33	1.49	0.50	0.61	4.74	2.46
33	CR 2499	5	39.85	13.90	5.37	0.22	5.59	0.41	0.03	0.65	0.76	0.82	1.06	0.80	0.72
34	OYR 69	7	30.65	11.28	1.29	0.09	1.38	0.14	0.03	1.20	1.89	0.21	0.54	6.01	3.51
35	Pant Dhan 4	9	17.63	8.75	0.13	0.07	0.20	0.03	0.02	1.12	2.75	0.19	0.41	6.07	6.79
36	Ananga	9	18.18	10.76	0.16	0.12	0.28	0.09	0.02	1.34	2.80	0.29	0.51	4.66	5.55
37	PMK-1	3	48.44	12.87	5.51	0.47	5.98	0.62	0.08	0.77	0.56	1.26	1.70	0.61	0.33
38	PRR103	5	45.65	13.13	3.17	0.27	3.43	0.35	0.04	0.70	0.73	0.95	1.13	0.74	0.65
39	PRR117	9	21.83	8.85	1.10	0.07	1.17	0.07	0.02	1.51	1.87	0.78	0.40	1.92	4.68
40	SKAU 220	9	23.81	9.61	2.02	0.10	2.11	0.07	0.02	1.04	1.83	0.72	0.84	1.46	2.20
41	Bhadrakali	7	21.75	8.50	0.47	0.06	0.52	0.03	0.02	1.27	2.25	0.82	0.46	1.56	4.91
42	NDR 97	5	57.81	19.60	4.14	0.49	4.63	0.35	0.05	0.79	0.58	1.26	0.96	0.63	0.60
43	NDR 359	5	48.83	14.10	3.69	0.39	4.08	0.38	0.04	0.95	0.82	1.27	1.39	0.75	0.59
44	Indravati	9	42.80	10.75	1.12	0.09	1.20	0.07	0.02	1.36	2.81	0.63	1.05	2.15	2.70
45	Pant Dhan 18	9	18.88	8.90	0.18	0.08	0.26	0.06	0.02	1.10	2.92	0.75	0.95	1.47	3.10
46	PRR121	5	43.63	20.03	3.65	0.35	3.99	0.42	0.05	0.74	0.62	0.88	1.00	0.85	0.49
47	PRR104	9	34.78	7.68	0.67	0.08	0.75	0.06	0.02	1.35	1.85	0.50	0.80	2.72	2.32
48	PRR115	5	43.73	15.15	4.20	0.41	4.60	0.46	0.03	0.84	0.56	1.25	0.90	0.67	0.62
49	PRR120	5	44.28	14.95	4.01	0.33	4.34	0.40	0.04	0.84	0.89	1.23	1.50	0.68	0.59
50	Pusa 1301	9	12.80	9.98	0.30	0.09	0.39	0.01	0.02	0.69	1.55	0.26	0.31	2.65	5.08

STS,salt tolerance score; SL, shoot length (cm); RL, root length (cm); SFW, shoot fresh weight (g); RFW, root fresh weight (g); SEW, seedling weight (g); SDW, shoot dry weight (g); RDW, root dry weight (g); RNC, root Na^+ content (mmol/g); SNC, shoot Na^+ content (mmol/g); RKC, root K^+ content (mmol/g); SKC, shoot K^+ content (mmol/g); RNK, root Na^+/K^+ ratio; SNK, shoot Na^+/K^+ ratio.

Table S1. Con.td

No.	Genotype	STS	SL	RL	SFW	RFW	SEW	SDW	RDW	RNC	SNC	RKC	SKC	RNK	SNK
51	Seond Basmati	3	67.35	18.55	6.24	0.79	7.03	0.60	0.05	0.71	0.58	1.10	1.49	0.65	0.39
52	Urvashi	5	54.20	15.10	6.13	0.56	6.69	0.50	0.04	0.85	0.84	1.18	1.44	0.72	0.58
53	Pant Dhan 16	7	35.88	13.88	2.24	0.07	2.31	0.13	0.02	2.31	1.90	0.61	0.48	3.83	4.00
54	Pusa 1490-03	7	13.15	7.85	0.24	0.05	0.30	0.02	0.02	2.23	1.92	0.61	0.50	3.65	3.88
55	JR 75	9	15.78	8.68	1.23	0.09	1.32	0.14	0.02	1.52	1.94	0.58	0.44	2.63	4.41
56	Pant Sugandh Dhan 15	9	26.95	12.75	1.34	0.09	1.43	0.10	0.02	0.98	1.74	0.46	0.42	2.16	4.14
57	Mahanadi	9	22.13	11.73	0.57	0.09	0.66	0.05	0.02	1.69	1.82	0.16	0.34	10.50	5.36
58	CO-37	5	44.96	18.10	4.32	0.41	4.73	0.40	0.04	0.71	0.78	1.00	1.01	0.71	0.77
59	Sumati	9	24.58	10.73	0.56	0.08	0.65	0.06	0.02	2.73	2.85	0.30	0.36	9.10	7.92
60	HUR-105	5	45.20	15.05	4.47	0.42	4.89	0.54	0.05	0.69	0.53	0.95	0.91	0.73	0.58
61	Manaswini	3	52.55	15.52	6.44	0.69	7.13	0.51	0.06	0.65	0.51	1.17	1.90	0.55	0.27
62	CN-1268-7	5	46.30	18.70	4.41	0.46	4.87	0.37	0.04	0.79	0.84	0.99	1.50	0.79	0.56
63	WGL-23985	7	15.93	9.00	0.16	0.07	0.22	0.03	0.02	1.07	1.65	0.27	0.82	3.96	2.01
64	Sharbati	5	55.78	14.13	4.33	0.55	4.88	0.53	0.05	1.08	0.83	1.60	1.31	0.68	0.64
65	Sambha Mashuri (BPT 5204)	9	32.00	10.00	0.67	0.09	0.75	0.07	0.02	1.73	1.97	0.52	0.56	3.37	3.51
66	UPRI-2003-15	9	26.15	7.38	0.51	0.08	0.58	0.04	0.02	1.16	2.25	0.60	1.00	1.93	2.25
67	Chittimutyalu	9	19.95	11.25	0.34	0.09	0.43	0.02	0.02	1.69	2.83	0.21	0.88	8.33	3.23
68	ASD19	9	16.88	6.50	0.49	0.06	0.54	0.03	0.02	2.70	3.70	0.83	1.49	3.26	2.48
69	Super Basmati	7	19.95	11.85	0.32	0.10	0.41	0.03	0.01	0.72	2.51	0.22	0.51	3.45	4.99
70	UPRVS-8-26	7	24.90	11.75	0.47	0.11	0.58	0.10	0.02	1.59	1.69	0.54	0.41	2.92	4.15
71	Shah Pasand	3	52.01	12.58	5.51	0.33	5.84	0.49	0.08	0.86	0.46	1.27	1.81	0.68	0.25
72	B 6144-MR-6-0-0	7	32.38	10.25	0.89	0.09	0.98	0.05	0.02	1.15	2.34	0.61	0.90	1.89	2.61
73	Chimbalate Basmati	9	31.80	7.38	0.99	0.09	1.07	0.05	0.03	2.97	3.27	0.63	0.37	4.74	8.85
74	Tilak Chandan	9	22.83	6.80	0.49	0.08	0.57	0.03	0.02	2.81	2.21	0.50	0.50	5.62	4.49
75	MR 219	7	34.38	11.25	0.86	0.10	0.96	0.14	0.02	1.24	1.50	0.65	0.44	1.92	3.41
76	Muskan	7	31.80	9.33	1.22	0.09	1.30	0.07	0.02	0.92	1.56	0.24	0.44	3.90	3.53

STS,salt tolerance score; SL, shoot length (cm); RL, root length (cm); SFW, shoot fresh weight (g); RFW, root fresh weight (g); SEW, seedling weight (g); SDW, shoot dry weight (g); RDW, root dry weight (g); RNC, root Na^+ content (mmol/g); SNC, shoot Na^+ content (mmol/g); RKC, root K^+ content (mmol/g); SKC, shoot K^+ content (mmol/g); RNK, root Na^+/K^+ ratio; SNK, shoot Na^+/K^+ ratio.

Table S1. Con.td

No.	Genotype	STS	SL	RL	SFW	RFW	SEW	SDW	RDW	RNC	SNC	RKC	SKC	RNK	SNK
77	JGL-3828	9	23.83	7.40	0.59	0.08	0.67	0.07	0.02	1.74	1.55	0.21	0.44	8.53	3.52
78	Sagar Samba	7	33.75	12.13	2.07	0.27	2.34	0.15	0.02	0.77	1.90	0.37	0.50	2.07	3.84
79	Kudrat-3	9	24.75	8.23	0.67	0.09	0.75	0.07	0.02	2.77	1.97	0.47	0.30	5.89	6.68
80	Ajaya (RR8585)	9	16.75	7.55	0.19	0.08	0.27	0.10	0.02	1.50	2.73	0.50	0.65	3.05	4.24
81	Swarna Sub1	7	35.75	13.15	0.75	0.12	0.87	0.15	0.02	1.47	2.14	0.76	0.74	1.92	2.89
82	Arupathaam Kururai	9	17.25	9.20	1.95	0.17	2.12	0.14	0.03	1.87	3.23	0.71	1.03	2.68	3.14
83	CO-51	7	17.05	3.75	0.13	0.08	0.20	0.03	0.02	1.51	2.14	0.30	0.45	5.07	4.76
84	CO-50	5	55.91	15.38	5.56	0.45	6.01	0.52	0.05	0.87	0.68	1.24	1.54	0.70	0.44
85	Karuppunel	5	54.70	17.80	5.10	0.61	5.70	0.50	0.05	0.72	0.77	0.97	1.25	0.61	0.61
86	Improved Shambha Mashuri	7	34.10	8.00	0.67	0.17	0.84	0.07	0.02	1.44	1.41	0.57	0.25	2.55	5.64
87	Sabour Surbhit (RAU 3036)	9	32.63	9.25	0.79	0.08	0.86	0.06	0.02	1.16	2.12	0.46	0.80	2.54	2.67
88	Pusa Basmati 1460	5	44.43	15.10	5.60	0.46	6.05	0.47	0.04	1.36	0.93	1.72	1.20	0.79	0.77
89	Type-3	9	24.75	7.50	0.84	0.12	0.95	0.07	0.02	2.31	1.49	0.78	0.52	2.97	2.89
90	Cotton Dora Sannalu (MTU 1010)	9	29.00	8.40	0.24	0.10	0.33	0.10	0.02	1.82	3.01	0.57	0.70	3.19	4.30
91	NDR 9830144	9	16.15	7.93	0.36	0.09	0.45	0.12	0.02	1.31	1.81	0.57	0.71	2.31	2.55
92	Jhulhat	7	25.85	9.75	0.64	0.10	0.74	0.12	0.02	0.61	1.65	0.49	0.67	1.26	2.48
93	Pratikshya	5	47.70	16.05	4.27	0.36	4.63	0.46	0.03	0.85	0.75	1.17	1.02	0.72	0.73
94	Pusa 33	5	52.88	17.35	6.44	0.55	6.99	0.42	0.05	0.77	0.82	1.12	0.97	0.69	0.85
95	Swarna (MTU 7029)	7	36.75	10.00	1.03	0.10	1.13	0.11	0.02	1.40	1.81	0.67	0.84	2.09	2.14
96	IRAT 240 (IREM950)	9	19.80	8.42	0.67	0.10	0.77	0.05	0.03	2.26	1.85	1.04	0.29	2.17	6.53
	Mean		35.47	12.44	2.51	0.24	2.75	0.22	0.03	1.30	1.56	0.80	0.92	2.43	2.64
	Minimum		12.80	3.75	0.07	0.05	0.12	0.01	0.01	0.53	0.46	0.16	0.24	0.34	0.25
	Maximum		67.35	24.20	9.25	0.85	9.57	0.62	0.09	2.99	3.72	1.82	1.96	10.50	8.85
	SD		14.39	4.18	2.40	0.21	2.59	0.19	0.02	0.61	0.79	0.41	0.51	2.10	2.09
	SE_Mean		1.04	0.30	0.17	0.02	0.19	0.01	0.00	0.04	0.06	0.03	0.04	0.15	0.15

STS,salt tolerance score; SL, shoot length (cm); RL, root length (cm); SFW, shoot fresh weight (g); RFW, root fresh weight (g); SEW, seedling weight (g); SDW, shoot dry weight (g); RDW, root dry weight (g); RNC, root Na^+ content (mmol/g); SNC, shoot Na^+ content (mmol/g); RKC, root K^+ content (mmol/g); SKC, shoot K^+ content (mmol/g); RNK, root Na^+/K^+ ratio; SNK, shoot Na^+/K^+ ratio; SD, Standard deviation; SE_standard error of mean.

Table S2. Details of germplasm used in the current study.

S.No.	Genotype	Parentage	Year	Duration	Ecosystem	Adaptation area
1	FL478	IR29/Pokkali	1997	85-90	IrU	-
2	CR 2461-9	-	-	-	IrL	OD
3	UPRI 2003-45	IR00A102/ IR66452-179-2-6-4-1	-	-	-	-
4	PNR 381	Tainan 3 mutant/ Basmati370	1992	85-105	RfU	WB
5	Pusa Sugandh 3	Pusa 1238-1/ Pusa 1238-81-6	2002	125-130	IrL	PB, HR, DL, UK, UP
6	Early Samba (RNRM 7)	Mutant of BPT5204	-	130-135		AP
7	Pant Dhan 19	BG 132/ UPRI 95-141	2007	130	IrL	PB, HR, GJ, MH
7	CSR 27	Nona Bokra/ IR565-33-2	1998	125	IrL	AI
8	Basmati 370	Selection from Dehraduni Basmati	1973	150	IrL	HR
9	NDR 8015-1	IR 72014-8 / NDR 1-1-1 B 53	-	-	-	UP
10	Samanta	T90/ IR8// Vikram/ Siam/// Mahsuri		140		OD
11	Tapaswini	Jagannath/ Mahsuri	1997	135	IrM	
12	Birupa	ADT 27/ IR 8 // Annapurna	1992	-	RfU	OD
13	Bhubana	-	1988	-	IrM	OD
14	Pusa Basmati 1121	Pusa 614-1-2/ Pusa 614-2-4-3	2003	140-145	-	PB, HR, UP, UK
15	Tompha Khau	Landrace	-	-	-	MN
16	Chandana	Sona/Manoharasali	1989	145	Ir	AP
17	VLT 6	-	-	-	-	-
18	VOH-PCR-3110	-	-	-	-	-
19	BJ 1	-	-	-	Ir	Bangladesh
20	Kamlesh	-	-	-	IrL	-
21	Narendra Usar Dhan III	Lung YAI 148 / IR 9125-209-2-2-2-1 // IR 1872-27-3-1	2000	125-140	IrSA	UP
22	Narendra Usar Dhan II	IR1814/IR1366- 120 -3- 1//IR1539- 37-3-1	1997	130	IrSA	UP
23	WGL 14	BPT 5204 / ARC 5984 // BPT 3291	2005	135-140	IrM	AP
24	Khara Munga	Landrace	-	-	-	
25	Apo	UPL RI 5/ IR 12979-24-1	2012	120	RfU	OD, CH

Aro, aromatic; IrL, irrigated lowland; RfU, rainfed upland; HlR, hill rice; Ir, irrigated; IrM, irrigated medium; IrU, irrigated upland; RfL, rainfed lowland; IrSA, irrigated saline alkaline; SwL, swampy lands; CoS, coastal saline; - information not available; AI, all India; AP, Andhra Pradesh; AS, Assam; BH, Bihar; CH, Chattisgarh; DL, Delhi; GJ, Gujarat; HP, Himachal Pradesh; KA, Karnataka; KL, Kerala; OD, Odisha; MN, Manipur; MH, Maharashtra; MP, Madhya Pradesh; PB, Punjab; JK, Jammu & Kashmir; HR, Haryana; WB, West Bengal; CH, Chhattisgarh; PY, Pondicherry; TG, Telangana; UK, Uttarakhand; UP, Uttar Pradesh; -, no information available.

Table S2. Con.td

S.No.	Genotype	Parentage	Year	Duration	Ecosystem	Adaptation area
26	CSR 23	IR64//IR4630-22-2-5-1-3/IR9764-45-2-2	2004	130-135	IrSA	MH, GJ, KE, TN, WB
27	Pusa 1342	P1154-2/ P1201-92-11	-	-	Aro	-
28	Pant Dhan 10	IR 32 // Mahsuri / IR 28	1993	125	IrM	UP
29	UPRI 2003-18	-	-	-	-	-
30	UPRI 2003-24	-	-	-	-	-
31	Sarjo 52	T(N)1/Kashi	1982	130-133	IrU	UP
32	Nagina 12	Selection from landrace	-	-	-	UP
33	CR 2499	BG 90-2/IR 67962-84-2-2-2	-	-	IrL	OD
34	OYR 69	-	-	-	-	KA
35	Pant Dhan 4	IR 262/ Remadja	1983	128-130	IrL	UP, UK
36	Ananga	Kumar (T 90/ IR 8) // CR 57-49	1989	120	IrL	AI
37	PMK 1	CO 25/ ADT 31	1982	120-125	RfL	TN
38	PRR 103	-	-	-	-	-
39	PRR 117	-	-	-	-	-
40	SKAU 220	-	-	-	-	JK
41	Bhadrakali	Phalguna/ IR 36	1994	130-135	IrM	AP
42	NDR 97	N22/ Ratna	1991	100	RfU	UP
43	NDR 359	BG-90-2-4/ 08677	-	-	Ir	UP
44	Indravati	IR 56/ OR 142-99		150	IrL	OD
45	Pant Dhan 18	IR 25394-3-57// RD 23// IR 27316-96/// SPRLR 77205-3-2	2007	105-135	IrL	BH, WB, OD, CH, AP, KA, TN
46	PRR 121	-	-	-	IrL	PB
47	PRR 104	-	-	-	-	-
48	PRR 115	-	-	-	-	-
49	PRR 120	-	-	-	-	-
50	Pusa 1301	Improved Sabarmati/ Khalsa 7	-	135	Aro	-
51	Seond Basmati	Landrace	-	-	Aro	HP
52	Urvashi	-	-	135	-	-

Aro, aromatic; IrL, irrigated lowland; RfU, rainfed upland; HlR, hill rice; Ir, irrigated; IrM, irrigated medium; IrU, irrigated upland; RfL, rainfed lowland; IrSA, irrigated saline alkaline; SwL, swampy lands; CoS, coastal saline; - information not available; AI, all India; AP, Andhra Pradesh; AS, Assam; BH, Bihar; CH, Chattisgarh; DL, Delhi; GJ, Gujarat; HP, Himachal Pradesh; KA, Karnataka; KL, Kerala; OD, Odisha; MN, Manipur; MH, Maharashtra; MP, Madhya Pradesh; PB, Punjab; JK, Jammu & Kashmir; HR, Haryana; WB, West Bengal; CH, Chhattisgarh; PY, Pondicherry; TG, Telangana; UK, Uttarakhand; UP, Uttar Pradesh; -, no information available.

Table S2. Con.td

S.No.	Genotype	Parentage	Year	Duration	Ecosystem	Adaptation area
53	Pant Dhan 16	BG380/BG367-4	2001			
54	Pusa 1490-03	Heibao/ P1302-3-3-1-10-02-1	-	-	Aro	-
55	JR 75	IR20/ L14// BSJ205	-	80-85	RfU	MP
56	Pant Sugandh Dhan 15	Basmati 370/ Sudari// Behral/ Muskan 41	2002	145	IrL	UP
57	Mahanadi	IR 19661-131/ Savitri	-	150	IrL	OD
58	CO 37	TN 1/ CO 29	1978	115	IrL	TN, PY
59	Sumati	Chandan / Pakistan Basmati	2002	140	-	-
60	HUR105	Mutant of MPR7-2		130-135	Ir	EI
61	Manaswini	Swarna / Lalat	2008	125-132	IrL, Rf	OD
62	CN 1268-7	-	-	-	IrL	WB
63	WGL 23985	Kavya / AC20	2009	Early	IrM	AP
64	Sharbati	Landrace	-	-	-	-
65	BPT 5204 (Sambha Mahsuri)	GEB 24/ T(N) 1// Mahsuri	1979	-	IrL	AP
66	UPRI 2003-15	IR00A102/ IR66452-179-2-6-4-1	-	-	-	-
67	Chittimuthylu	Landrace	-	-	Aro	AP
68	ASD 19	Lalnakanda/ IR 30	1997	120-132	IrL	TN
69	Super Basmati	Basmati 320/ IR 661	2004	145	IrL	PB
70	UPRVS 8-26	-	-	-	-	-
71	Shah Pasand	Landrace	-	-	Aro	
72	B 6144-MR-6-0-0	Landrace	-	-	HlR	West Africa
73	Chimbalate Basmati	Landrace	-	-	-	JK
74	Tilak Chandan	Landrace	2009	-	-	UK
75	MR 219	MR 137/MR 151	-	-	-	Malaysia
76	Muskan	Landrace	-	-	-	JK
77	JGL 3828	Samba Mahsuri/ Aganni	2009	135-150	IrL	TG
78	Sagar Samba	IR 8 / Siam 29 // IR 8 / PTB 21	1993	150	RfL	AP
79	Kudrat 3	Selection from landrace	-	120-135	-	UP

Aro, aromatic; IrL, irrigated lowland; RfU, rainfed upland; HlR, hill rice; Ir, irrigated; IrM, irrigated medium; IrU, irrigated upland; RfL, rainfed lowland; IrSA, irrigated saline alkaline; SwL, swampy lands; CoS, coastal saline; - information not available; AI, all India; AP, Andhra Pradesh; AS, Assam; BH, Bihar; CH, Chattisgarh; DL, Delhi; GJ, Gujarat; HP, Himachal Pradesh; KA, Karnataka; KL, Kerala; OD, Odisha; MN, Manipur; MH, Maharashtra; MP, Madhya Pradesh; PB, Punjab; JK, Jammu & Kashmir; HR, Haryana; WB, West Bengal; CH, Chhattisgarh; PY, Pondicherry; TG, Telangana; UK, Uttarakhand; UP, Uttar Pradesh; -, no information available.

Table S2. Con.td

S.No.	Genotype	Parentage	Year	Duration	Ecosystem	Adaptation area
80	Ajaya (RR 8585)	IET4141/ CR 98-7216	1992	130-135	IrM	AS, GO, PY
81	Swarna Sub1	Swarna 3/ IR 49830-7-1-2-3	2009	140	-	AI
82	Arupathaam Kuruvai	Landrace	-	60	IrL	TN
83	CO 51	ADT 43/ RR 272 – 1745	2005	105-110	IrL	TN
84	CO 50	CO 43 / ADT 38	2010	130-135	IrM	TN
85	Karuppunel	Landrace	-	-	-	TN
86	Improved Samba Mahsuri	Samba Mahsuri*4/SS1113	208	140-145	IrL	AP, TN, GH
87	Sabour Surbhit (RAU 3036)	Mutant of Rajendra Suhasini	2017	92-95	IrM	BH
88	Pusa 1460	Pusa Basmati 1//Pusa Basmati 1/lRBB 55	2007	-	Aro	-
89	Type 3	Landrace	-	-	-	UP
90	Cotton Dora Sannalu (MTU 1010)	Krishnaveni/ IR-64	-	120-125	IrM	AP
91	NDR 9830144	IR 60185-B-25-2-2 / IR 57519-PMI-5-3-2- 2 // IR 55008-10-3-3 - 3-3	2008	140-145	IrL	UP
92	Jhulhat	Landrace	-	-	-	-
93	Pratikshya	Swarna /IR 64	2006	142	IrL, RfL	OD
94	Pusa 33	Ratna/Pusa 2-21	-	-	-	PB, HR, UP, UK
95	Swarna (MTU 7029)	Vasista/ Mahsuri	1987	150	IrL	OD, AP
96	IRAT 240 (IREM950)	Mutant of IAC25	1980	-	-	Guyana

Aro, aromatic; IrL, irrigated lowland; RfU, rainfed upland; HlR, hill rice; Ir, irrigated; IrM, irrigated medium; IrU, irrigated upland; RfL, rainfed lowland; IrSA, irrigated saline alkaline; SwL, swampy lands; CoS, coastal saline; - information not available; AI, all India; AP, Andhra Pradesh; AS, Assam; BH, Bihar; CH, Chattisgarh; DL, Delhi; GJ, Gujarat; HP, Himachal Pradesh; KA, Karnataka; KL, Kerala; OD, Odisha; MN, Manipur; MH, Maharashtra; MP, Madhya Pradesh; PB, Punjab; JK, Jammu & Kashmir; HR, Haryana; WB, West Bengal; CH, Chhattisgarh; PY, Pondicherry; TG, Telangana; UK, Uttarakhand; UP, Uttar Pradesh; -, no information available.

Table S3. The chemical composition of modified Yoshida nutrient solution used in the study. The nutrient concentrations are the same as that of the original composition (Yoshida 1976). The details of the preparation of stock and culture solutions are also given.

Element solution*	Reagent	Formula	Quantity for stock		Nutrients		Culture	
			g/10L	g/L	%	g/L	ppm	Stock (ml)/4 L
**Stock A**								
K+N	Potassium nitrate	KNO_3	567.3	56.73	38.67 K<br>13.85 N	21.94 K<br>7.86 N	40	5.0
N	Ammonium sulphate	$(NH_4)_2SO_4$	1138.0	113.80	21.20 N	24.13 N	40	5.0
K+P	Potassium dihydrogen phosphate	KH_2PO_4	351.5	35.15	22.76 P<br>28.73 K	8.00 P<br>10.10 K	10	5.0
**Stock B**								
Ca	Calcium chloride	$CaCl_2$	886.0	88.60	36.11	32.00	40	5.0
Mg	Magnesium sulphate	$MgSO_4.7H_2O$	3240.0	324.00	9.86	31.95	40	5.0
**Stock C**								
Mn	Manganese chloride	$MnCl_2.2H_2O$	15.0	1.50	33.94	0.51	0.5	5.0
Mo	Ammonium molybdate	$(NH_4)_6Mo_7O_{24}.4H_2O$	0.74	0.074	54.34	0.04	0.05	
B	Boric Acid	H_3BO_3	9.34	0.934	17.48	0.16	0.2	
Zn	Zinc sulphate	$ZnSO_4.7H_2O$	0.35	0.035	22.74	0.008	0.01	
Cu	Copper sulphate	$CuSO_4.5H_2O$	0.31	0.031	25.45	0.008	0.01	
Fe	Ferrous sulphate[§]	$FeSO_4.7H_2O$	79.20	7.92	20.09	1.60	2	
	Citric acid (monohydrate)[¶]		119.0	11.9				

* Constitute the culture solution prior to use in required quantities to avoid wastage.

§ Ferrous sulphate is to be constituted fresh every time in required quantities. Try avoiding storage.

¶ Citric acid is to be added to freshly prepared $FeSO_4$ solution to stabilise it from getting oxidised to toxic Fe(III) form.

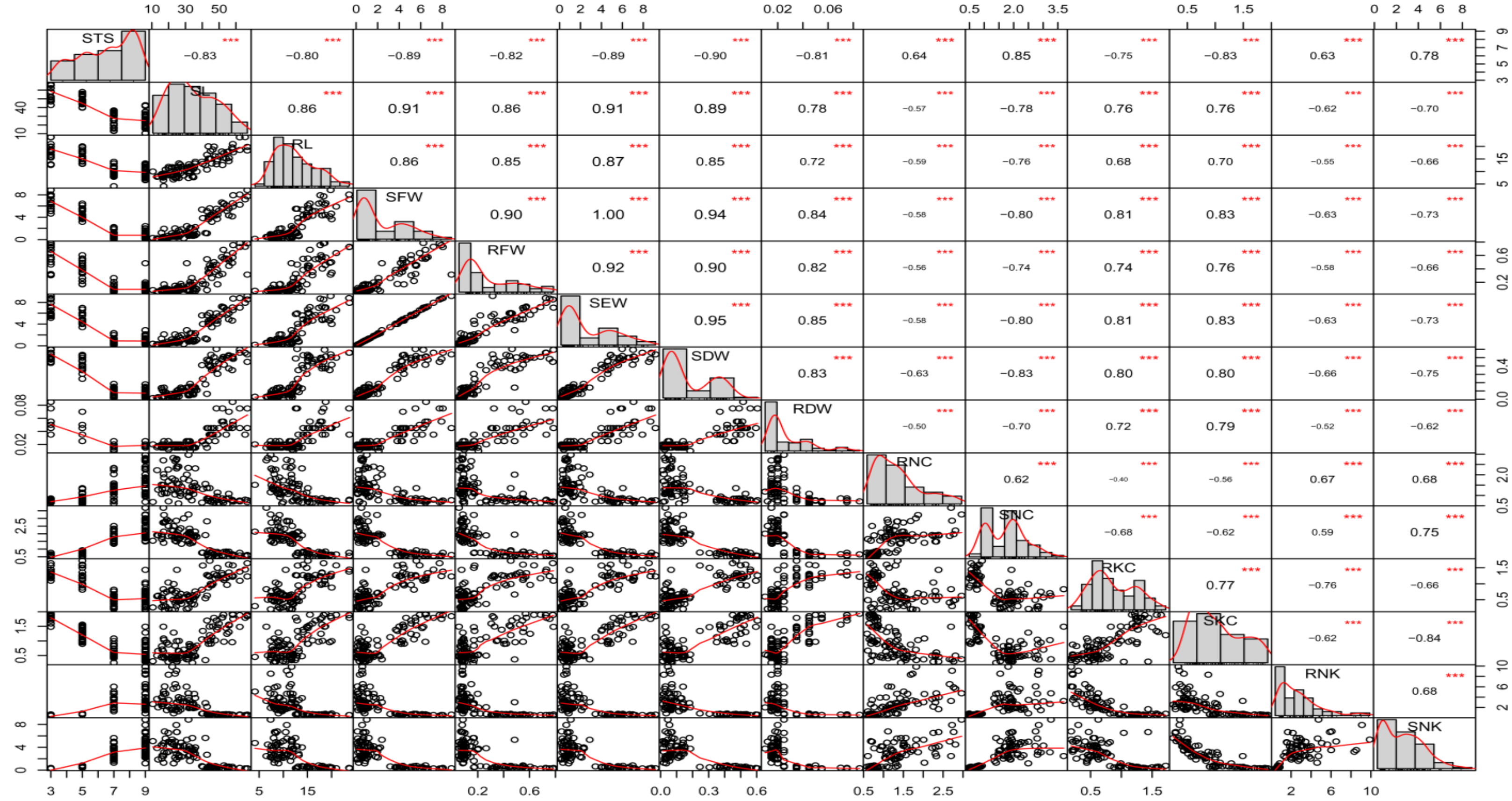

Supplementary Figure 1. Correlation between the traits for seedling stage salinity tolerant. STS, Salinity tolerance score; SL, shoot length (cm); RL, root length (cm); SFW, shoot fresh weight (g); RFW, root fresh weight (g); SEW, seedling weight (g); SDW, shoot dry weight (g); RDW, root dry weight (g); RNC, root Na^+ content (mmol/g); SNC, shoot Na^+ content (mmol/g); RKC, root K^+ content (mmol/g); SKC, shoot K^+ content (mmol/g); RNK, root Na^+/K^+ ratio; SNK, shoot Na^+/K^+ ratio.
